# Metal–Organic-Framework-Based Optical Biosensors: Recent Advances in Pathogen Detection and Environmental Monitoring

**DOI:** 10.3390/s25165081

**Published:** 2025-08-15

**Authors:** Alemayehu Kidanemariam, Sungbo Cho

**Affiliations:** 1 Department of Electronic Engineering, Gachon University, Seongnam-si 13120, Republic of Korea; 2 Department of Semiconductor Engineering, Gachon University, Seongnam-si 13120, Republic of Korea; 3 Gachon Advanced Institute for Health Science & Technology, Gachon University, Incheon 21999, Republic of Korea

**Keywords:** metal–organic frameworks, optical biosensors, wearable biosensors, environmental monitoring, colorimetric sensors, fluorescence sensing, luminescent metal–organic frameworks, pathogen detection, heavy metal sensing, real-time detection

## Abstract

Metal–organic frameworks (MOFs) have emerged as highly versatile materials for the development of next-generation optical biosensors owing to their tunable porosity, large surface area, and customizable chemical functionality. Recently, MOF-based platforms have shown substantial potential in various optical transduction modalities, including fluorescence, luminescence, and colorimetric sensing, enabling the highly sensitive and selective detection of biological analytes. This review provides a comprehensive overview of recent advancements in MOF-based optical biosensors, focusing on their applications in pathogen detection and environmental monitoring. We highlight key design strategies, including MOF functionalization, hybridization with nanoparticles or dyes, and integration into microfluidic and wearable devices. Emerging methods, such as point-of-care diagnostics, label-free detection, and real-time monitoring, are also discussed. Finally, the current challenges and future directions for the practical deployment of MOF-based optical biosensors in clinical and field environments are discussed.

## 1. Introduction

The global burden of infectious diseases and environmental pollution continues to challenge public health, food and water safety, and ecological stability [1,2]. Pathogenic microorganisms such as bacteria, viruses, and fungi are responsible for widespread morbidity and mortality, whereas contaminants such as heavy metals, pesticides, and organic pollutants threaten ecosystems and human well-being [3,4,5]. Early, accurate, and on-site detection of these biological and chemical hazards is critical for controlling disease outbreaks, ensuring environmental compliance, and supporting timely intervention strategies [6]. However, conventional analytical approaches, including culture-based microbiological assays, polymerase chain reaction (PCR), enzyme-linked immunosorbent assay (ELISA), atomic absorption spectroscopy (AAS), and high-performance liquid chromatography, though highly reliable in laboratory settings, often fall short in field applications due to their reliance on expensive instrumentation, time-intensive workflows, and the need for skilled personnel [7,8].

In this context, biosensors have emerged as transformative tools that combine biological recognition elements with signal transduction mechanisms to detect target analytes with high specificity and high sensitivity [9]. Among various types of biosensors, optical biosensors have gained considerable attention because their unique advantages [10]. These systems transduce molecular recognition events into optical signals typically via luminescence-based mechanisms (such as fluorescence and chemiluminescence), surface plasmon resonance, or colorimetric changes, which are readily detectable often in real time [11,12]. Optical biosensors offer several benefits over electrochemical or piezoelectric biosensors, including rapid signal generation, minimal interference from sample matrices, and compatibility with visual and smartphone-based detection platforms [13]. Their capacity for multiplexing, miniaturization, and non-contact readouts further supports their use in decentralized, point-of-care (POC), and resource-limited settings [14].

The effectiveness of optical biosensors in pathogen detection is largely attributed to their ability to provide rapid, highly sensitive, and selective analyses with minimal sample processing [15]. By targeting pathogen-specific biomarkers such as nucleic acids, proteins, lipopolysaccharides, and whole cells, biosensors can discriminate between closely related species and strains [16]. Fluorescence-based sensors, for instance, can exploit hybridization probes, aptamers, or antibody–antigen interactions to trigger measurable emission changes upon binding to the target analyte [17,18]. Similarly, colorimetric biosensors enable naked-eye detection through visually discernible color shifts induced by enzyme-catalyzed reactions and nanoparticle aggregation [19]. Such attributes not only enable early diagnosis and environmental warning systems, but also facilitate continuous real-time monitoring, which is particularly important during disease outbreaks or exposure to dynamic pollution sources [20].

Despite these advantages, the performance of optical biosensors is heavily dependent on the materials used for signal generation, amplification, and biorecognition [21]. This has led to intensive research into advanced materials capable of enhancing sensitivity, selectivity, and operational stability [22]. Among these, metal–organic frameworks (MOFs) have emerged as a highly promising class of materials for next-generation biosensor development [23]. MOFs are crystalline porous materials composed of metal ions or clusters coordinated with organic linkers, yielding tunable three-dimensional architectures [24]. They exhibit exceptionally high surface areas (often exceeding 5000 m^2^/g), adjustable pore sizes, and a high degree of chemical modularity [25]. These features render MOFs uniquely suited for hosting functional molecules, facilitating molecular interactions, and enhancing signal transduction in optical biosensing.

The intrinsic and tunable optical properties of MOFs further contribute to their applicability in this field [26]. Some MOFs exhibit intrinsic luminescence owing to the electronic transitions of metal clusters or organic linkers, whereas others serve as protective carriers for fluorescent dyes, quantum dots, or nanoparticles, preventing photobleaching and quenching [27]. MOFs can also act as signal amplifiers through mechanisms such as fluorescence resonance energy transfer (FRET, is a mechanism in which energy is nonradiatively transferred from an excited donor fluorophore to a nearby acceptor molecule), photoinduced electron transfer (PET, is a process in which an excited molecule, often a fluorophore, transfers an electron to a nearby acceptor or receives one from a donor upon light excitation), and inner filter effects (IFE, occur when an absorbing species in a sample diminishes the intensity of excitation or emission light, which can artificially alter measured fluorescence signals), depending on their structural and electronic characteristics [28,29]. Moreover, the surface of MOFs can be readily functionalized with aptamers, deoxyribonucleic acid (DNA) strands, peptides, or antibodies, providing specificity for targeted biological or environmental analytes [30]. These combined properties enable the design of highly sensitive, selective, and versatile optical biosensors with low detection limits and excellent performance in complex sample matrices [31].

MOF-based optical biosensors have been successfully used to detect a wide range of pathogens, including *Escherichia coli*, *Staphylococcus aureus*, *Salmonella enterica*, and severe acute respiratory syndrome coronavirus 2 (SARS-CoV-2), as well as to monitor toxic environmental contaminants, such as Pb^2+^, Hg^2+^, Cr^6+^, organophosphates, and nitroaromatic compounds [32,33,34]. These sensors exhibit outstanding analytical performance with detection limits in the femtomolar to picomolar range, rapid response times, and strong resistance to interference [35]. The incorporation of MOFs into portable devices, paper-based sensors, microfluidic chips, and wearable devices has opened new avenues for decentralized monitoring, offering low-cost, user-friendly, and field-deployable sensing solutions [36,37]. The innovations are particularly valuable for epidemiological surveillance, water quality assessment, agricultural monitoring, and early warning systems in underserved regions [20,38].

This review provides a comprehensive examination of recent developments in MOF-based optical biosensors, focusing on their applications in pathogen detection and environmental monitoring. The fundamental principles of optical biosensing and the rationale for using MOFs as functional materials are outlined. The review then explores various design strategies including MOF functionalization, hybridization with nanomaterials, and structural tailoring for enhancing sensing performance. Representative examples across different optical modalities such as fluorescence, chemiluminescence, and colorimetry are discussed in detail. Special attention is given to emerging trends such as label-free detection, smartphone integration, wearable sensors, and real-time monitoring. Finally, the key challenges in the field, including MOF stability under physiological conditions, reproducibility, scalability, and regulatory acceptance, and future research directions for advancing the practical implementation of MOF-based optical biosensors are discussed. To ensure a thorough and representative overview, relevant studies were collected from Web of Science, Scopus, PubMed, and Google Scholar using keywords including “MOF,” “optical biosensor,” “pathogen detection,” and “environmental monitoring.” We focused on recent publication reporting clear performance metrics such as sensitivity, detection limits, and stability. Reviews, non-English articles, and studies without experimental data were excluded. Each study was carefully screened by title and abstract, followed by full-text review, ensuring that only high-quality representative work was included. Finally, the key challenges in the field, including MOF stability, reproducibility, scalability, regulatory acceptance, and practical deployment, are discussed, along with future research directions to advance the real-world implementation of MOF-based optical biosensors.

## 2. Fundamental Mechanisms Underpinning MOF-Based Optical Biosensors

**Scheme 1:**
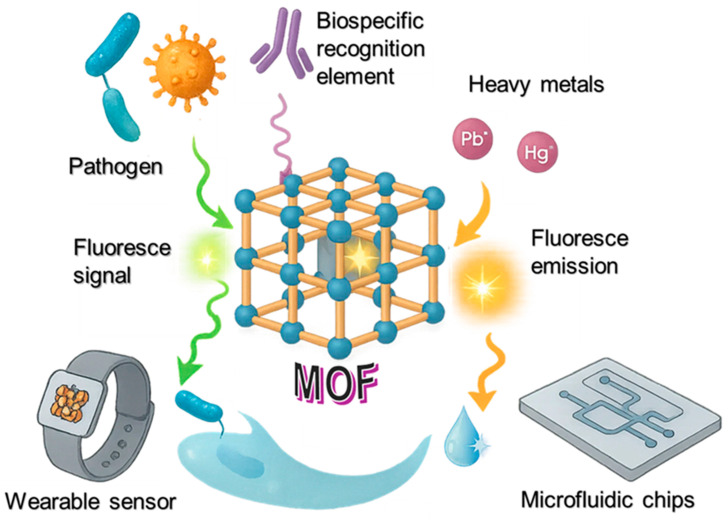
MOF-based optical biosensor detecting pathogens and heavy metals via fluorescence and color changes in environmental samples.

MOFs are an emerging class of crystalline hybrid materials composed of metal ions or clusters coordinated to organic linkers [39]. Their high surface area, structural order, and synthetic tunability make them ideal platforms for biosensing. In optical biosensing, MOFs offer unique benefits, including facile integration of optical reports (e.g., fluorophores), high analyte adsorption capacity, and modularity for engineering molecular recognition and transduction pathways. These properties underpin their roles in developing sensitive, selective, and versatile biosensing platforms (Scheme 1).

### 2.1. Structural and Chemical Tunability

One of the most advantageous features of MOFs is their structural and chemical tunability, which allows precise control over pore size, surface chemistry, and framework topology [40]. These properties are critical in biosensing, governing analyte accessibility and interaction dynamics within the sensing matrix [41]. The organic linkers and metal nodes can be systematically modified or replaced by introducing functional groups or coordination sites tailored for specific targets such as nucleic acids, proteins, or small molecules [42]. Post-synthetic modification techniques ligand exchange, covalent grafting, and metal ion doping expand the chemical diversity and functional capabilities of MOFs, facilitating enhanced binding affinities and selective recognition [43].

**Figure 1:**
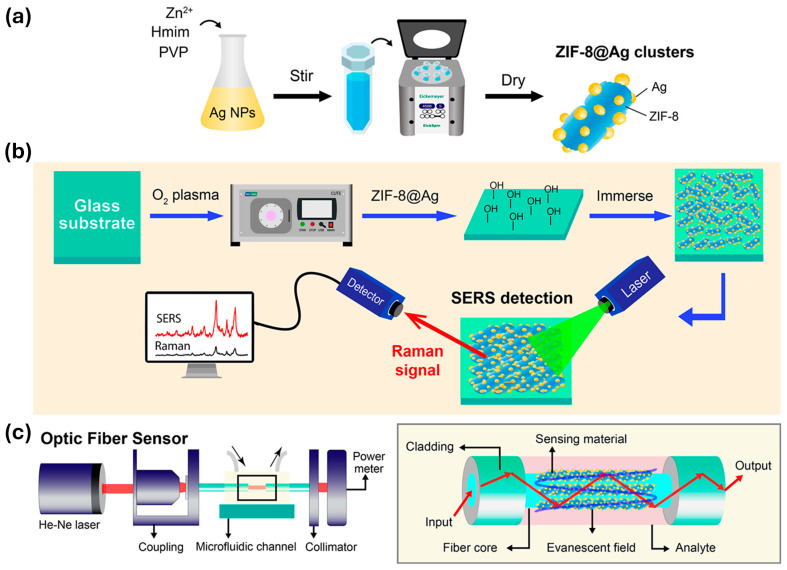
(**a**) Preparation of ZIF-8@Ag by mixing zinc nitrate, 2-methylimidazole, and PVP in Ag nanoparticle colloid. (**b**) Oxygen plasma treatment of glass substrates to form surface hydroxyl groups. (**c**) Functionalized 1 cm fiber with ZIF-8@Ag integrated into a microfluidic system for real-time sensing. Copyright 2025 ACS [44].

Building on this concept, Tran et al. present a ZIF-8@Ag heterostructure that leverages the porous and chemically tunable nature of ZIF-8 to stabilize silver nanoparticles and enhance analyte preconcentration [44]. As shown in Figure 1, the ZIF-8@Ag hybrid is prepared by mixing zinc nitrate, 2-methylimidazole, and PVP in a silver nanoparticle colloid. The synergistic interaction between the MOF structure and Ag nanoparticles boosts both electromagnetic and chemical signals, enabling ultrasensitive optical detection with high stability and reproducibility. The platform achieves ultralow detection limits for multiple analytes through SERS and fiber-optic localized surface plasmon resonance (LSPR) sensing. This work highlights how tuning MOF structure and chemistry can overcome common plasmonic sensor challenges, advancing optical biosensor performance and practical applications.

Expanding on the idea of multifunctionality, another study used a MOF-based gated nanoprobe integrated with hairpin DNA sequences to differentiate wild and vaccine strains of Brucella [45]. The MOF matrix acted as a protective scaffold for fluorophores, enabling precise and gated fluorescence release upon DNA hybridization. The dual-signal platform achieved a low limit of detection (LOD) of 6.4 × 10^−10^ M with over 90% detection accuracy, showcasing how framework integrity enhances selectivity and minimizes background interference. This highlights the dual role of MOFs in molecular protection and signal responsiveness.

In addition to biochemical detection, MOFs are increasingly being employed in early disease diagnosis. El-Sheikh et al. reported Zn-MOF nanoparticles (~118 nm) with tunable photoluminescence and thermal stability for detecting prostate-specific antigen (PSA) [46]. The sensor achieved a detection limit of 0.145 fg/mL over a linear range of 0.1 fg/mL to 20 pg/mL, demonstrating the feasibility of ultra-sensitive MOF-based clinical diagnostics.

While fluorescence-based detection dominates current applications, MOFs’ responsive frameworks are also exploited in colorimetric sensing. A notable example is the flexible MIL-53(Al) framework, which exhibits reversible structural transformations upon interaction with volatile organic compound (VOCs) like ketones, alcohols, and water [47]. These guest-induced changes in pore geometry influence its photoluminescence, allowing selective visual detection. Though not quantitative in terms of LOD, this qualitative shift reinforces the utility of framework responsiveness in environmental monitoring.

To bridge the gap between structural engineering and signal enhancement, future directions may involve combining MOFs with LSPR-based fiber optic biosensors for real-time and label-free detection [48]. Although not inherently MOF-based, LSPR systems could benefit significantly from the porous, tunable architecture of MOFs, enabling better molecular recognition, reduced noise, and signal amplification in portable diagnostic formats. As the above examples illustrate, structural and chemical tunability is fundamental for optimizing the sensitivity, selectivity, and responsiveness of MOF-based optical biosensors.

### 2.2. Optical Biosensing Strategies Using MOFs (e.g., Fluorescence, Colorimetry, SERS)

Transitioning from structure to function, MOFs’ ability to convert biological recognition events into measurable optical signals is key to their biosensing utility. The primary optical transduction mechanisms include fluorescence, colorimetric changes, and surface-enhanced Raman scattering (SERS) [49,50,51]. Among these, fluorescence is the most widely utilized, where MOFs either possess intrinsic luminescence or act as hosts for fluorophores and quantum dots. Signal modulation occurs via mechanisms such as FRET, PET, or IFE, enabling precise “turn-on” or “turn-off” sensing modes [52,53].

One exemplary system used a copper-based MOF (Cu-MOF) to develop a fluorescence biosensor for aflatoxin B1 detection [54]. The MOF simultaneously acted as a Cu(II) ion reservoir and antigen carrier. A target-induced click reaction triggered hybridization of fluorescent DNA strands, producing an amplified signal with an ultralow LOD of 0.48 pg/mL and a 670-fold improvement in sensitivity over conventional ELISA, emphasizing MOF-enabled transduction efficiency.

In addition to simple fluorescence signaling, light-responsive nanoscale MOFs (NMOFs) are gaining traction due to their ability to undergo optical changes in response to external stimuli [55]. Originally designed for cancer imaging, these NMOFs now serve biosensing roles by enabling spatiotemporally controlled optical outputs, further enriching the design space for smart biosensors.

The development of ratiometric fluorescence systems has improved biosensor reliability. Ratiometric fluorescence is a sensing approach that compares the intensity of two optical signals usually emitted at different wavelengths so as to minimize errors from environmental changes, instrument variability, or sample inconsistencies. The hybrid carbon nanodot (CD)/Co-MOF nanocoral served as a dual-channel sensor for alkaline phosphatase (ALP) detection [56]. The phosphate-triggered ligand exchange disrupted the MOF, releasing carbon dots (restoring fluorescence) and diminishing the second-order scattering signals. This allowed for a ratiometric optical output with an LOD of 0.6 mU/L and a linear range spanning six orders of magnitude, significantly enhancing precision and reproducibility.

Moreover, tuning the photophysical properties of MOFs provides a sensitive guest molecule loading capacity and an overall improved sensing efficiency. MOFs exhibit highly tunable photoluminescent properties arising from their metal nodes, organic linkers, or encapsulated guest species, making them excellent candidates for optical transduction in biosensors [57]. By judiciously selecting metal ions, organic ligands, and synthetic conditions, the fluorescence behavior of MOFs can be precisely engineered to enable the sensitive and selective detection of a wide range of analytes. Fluorescence-based MOF sensors typically operate via “turn-on” or “turn-off” mechanisms, with “turn-on” systems being particularly valuable for real-time sensing due to their enhanced signal-to-noise performance. These tunable optical responses facilitate the development of MOF-based sensors which are capable of detecting hazardous chemicals, biological molecules, and environmental contaminants, thus showcasing their potential as versatile platforms for fluorescence-based biosensing.

### 2.3. Functionalization Strategies for Specificity and Sensitivity

Although MOFs inherently offer high porosity and modularity, their specificity in biosensing is typically achieved through their functionalization. This includes covalent attachment or adsorption of recognition elements (e.g., aptamers, antibodies, peptides, and nucleic acids) and surface engineering via molecular imprinting or electrostatic interactions [58,59,60]. In one recent study, a MIL-53(Al)-NH_2_ framework was engineered to capture fragment crystallizable mannose-binding lectin (FcMBL)-conjugated elements for Bacillus cereus detection [61]. By combining aptamer-functionalized magnetic beads with this dual-recognition strategy, the MOF-based sensor achieved a remarkable LOD of 4 CFU/mL over a broad concentration range (20–2 × 10^8^ CFU/mL). This work highlights how tailored surface chemistry on the MOF significantly improves optical biosensing performance by integrating selective capture elements and fluorescent signaling within a single platform.

In another study, a gold-nanoparticle-loaded zeolitic imidazolate framework (ZIF)-MOF (Au NPs@ZIF) was functionalized with DNA aptamers to construct a ratiometric fluorescence sensor for detecting *S. aureus* [62]. The platform integrated methylene blue and ferrocene fluorophores, achieving intrinsic signal calibration and detecting *S. aureus* with a LOD of 1 CFU/mL and a broad linear range of 5 to 10^8^ CFU/mL. This highlights the impact of combining biorecognition and ratiometric signaling.

Complementing this strategy, a lateral flow immunoassay was developed using a bimetallic Au/Ir@Cu/Zn-MOF functionalized with anti-*S. aureus* antibodies [63]. The porous MOF structure enhanced signal intensity through nanoparticle dispersion and photothermal synergy, achieving a visual detection limit of 10^3^ CFU/mL, which is a 100-fold improvement over non-MOF probes. Beyond detection, this functionalized MOF exhibited bactericidal effects, expanding its application to combined diagnostics and therapy.

Functionalization also plays a central role in small molecule detection, as demonstrated by a dual-emission ratiometric sensor for adenosine triphosphate (ATP) [64]. The MOF core encapsulated Ru(bpy)_3_^2+^ as a stable fluorescent reference, while double-stranded deoxyribonucleic acid (dsDNA) on the surface allowed SYBR Green I (SGI) intercalation. ATP presence disrupted the DNA duplex, selectively quenching the SGI signal while preserving the Ru signal. This enabled an LOD of 0.63 µM. Embedded into agarose hydrogel and paired with a smartphone fluorescence reader, the sensor achieved real-time bacterial detection down to 10 CFU/mL in milk samples.

**Figure 2:**
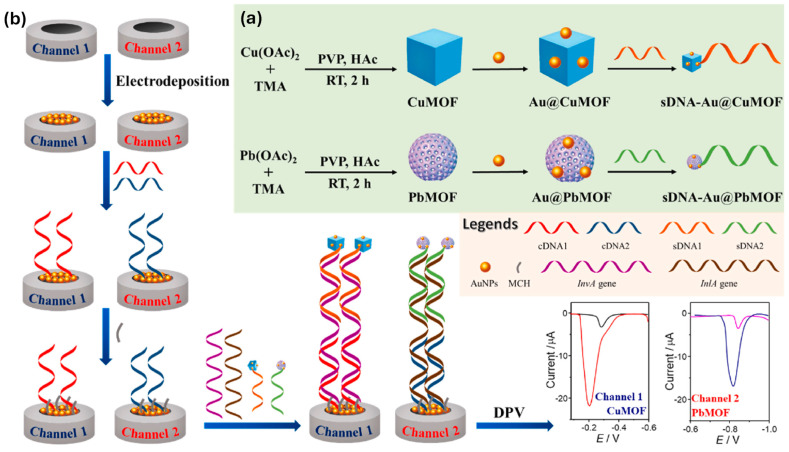
(**a**) Schematic illustration of the synthesis steps for CuMOF, PbMOF, Au@CuMOF, and Au@PbMOF. (**b**) Diagram showing the working principle of the dual-channel electrochemical biosensor for detecting *S. typhimurium* (invA gene) and *L. monocytogenes* (inlA gene). Copyright 2023 Elsevier [65].

Finally, MOF-based systems enable multiplexed biosensing, as shown in a dual-channel electrochemical biosensor using Au@CuMOF and Au@PbMOF nanotags [65]. This material were functionalized with DNA probes targeting *Salmonella typhimurium* (invA gene) and *Listeria monocytogenes* (inlA gene) (Figure 2), the platform produced distinct differential pulse voltammetry (DPV) signals for each ion (Cu^2+^ and Pb^2+^), achieving LODs of 2.33 CFU/mL for *S. typhimurium* and 6.61 CFU/mL for *L. monocytogenes*, with wide linear ranges and minimal cross-reactivity (Table 1).

These studies collectively highlight how surface functionalization strategies empower MOFs with high analyte specificity, minimal background interference, and versatile transduction formats for real-world applications in biosensing.

## 3. MOF-Based Electrochemical and Optical Biosensors for Pathogen Detection

The emergence of infectious diseases and antimicrobial resistance has heightened the need for rapid, sensitive, and portable pathogen detection technologies. MOFs, owing to their modularity, high porosity, and tunable optical/electrochemical properties, have gained prominence in biosensor design [66,67]. MOFs can be tailored for diverse transduction strategies including fluorescence, colorimetry, SERS, and electrochemical readouts [68]. This section reviews recent progress in MOF-based biosensors for microbial detection, focusing on three aspects: (i) microbial targets (bacteria, viruses, and fungi), (ii) detection strategies (label-free vs. labeled), and (iii) performance metrics such as sensitivity and LOD.

### 3.1. MOF-Based Biosensors for Microbial Detection

#### 3.1.1. Optical Biosensing Platforms

Recent advancements highlight the broad utility of MOF-based biosensors in targeting bacterial, viral, and fungal pathogens [69]. By tailoring the pore environment, surface functional groups, and framework composition, MOFs can be functionalized with aptamers, antibodies, and enzymes to achieve highly specific and sensitive detection. A notable example is a zirconium-based MOF functionalized with DNA aptamers for the fluorescence-based detection of *E. coli* O157:H7. The system exploited a fluorescence “turn-on” mechanism triggered by the specific binding between aptamer and pathogen, providing a highly selective and responsive readout [70]. Similarly, lanthanide-doped MOFs enabled time-resolved fluorescence sensing of *S. aureus*, minimizing autofluorescence and background interference. For viral targets, MOFs integrated with gold nanoparticles (AuNPs) and antibody tags were employed in a colorimetric platform for the detection of SARS-CoV-2 antigens. This system enabled visual detection within 15 min, making it a viable option for rapid point-of-care testing (POCT) [71]. Though less explored, fungal diagnostics have also benefited from MOF integration. In one case, MOFs functionalized with lectins or enzyme mimics enabled SERS-based fingerprinting of Candida albicans, demonstrating the material’s adaptability even in fungal biosensing [72].

**Figure 3:**
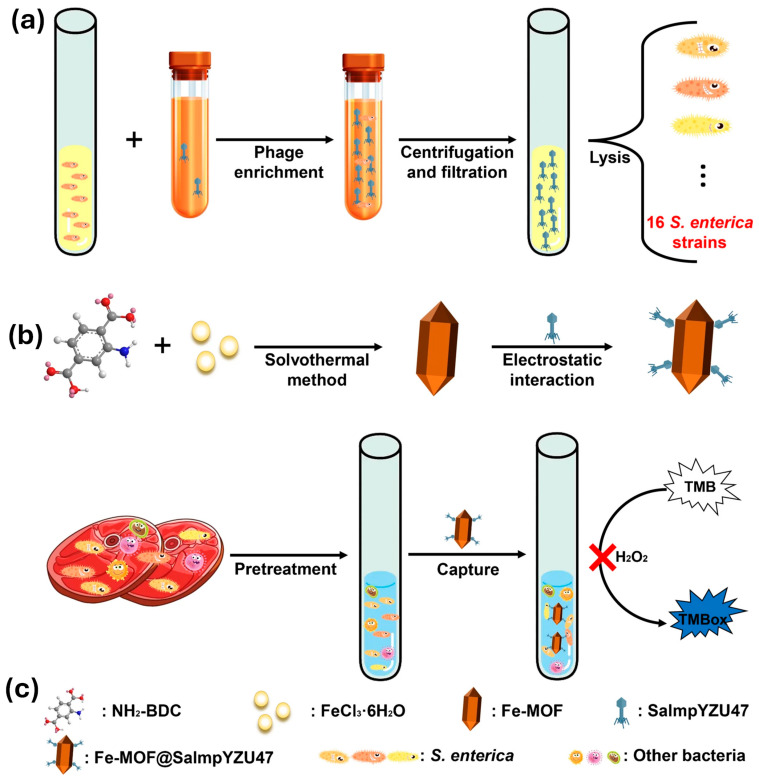
(**a**) Isolation and purification process of phage SalmpYZU47; (**b**) synthesis pathway of the Fe-MOF@SalmpYZU47 nanozyme; and (**c**) schematic of colorimetric detection of multiple *S. enterica* strains in food using the developed Fe-MOF@SalmpYZU47 platform. Copyright 2024 Springer Nature [73].

To broaden the scope to broader detection targets, a phage-functionalized Fe-MOF nanozyme (Fe-MOF@SalmpYZU47) was developed for the colorimetric detection of multiple *S. enterica* serovars (Figure 3) [73]. This platform used a broad-host-range bacteriophage (SalmpYZU47) immobilized on an Fe-based MOF, imparting both biorecognition and enzymatic mimicry. The peroxidase-like activity of the MOF nanozyme was selectively inhibited in the presence of *S. enterica*, yielding a colorimetric signal with a linear range of 1.0 × 10^2^ to 1.0 × 10^8^ CFU/mL and a detection limit of 11 CFU/mL. The system achieved recovery rates of 91.88–105.34% in the food samples, underscoring its practical utility.

#### 3.1.2. Electrochemical Biosensing Platforms

To further demonstrate point-of-care applicability, a portable and cost-effective visual biosensor was developed by integrating MOFs with a pregnancy test strip (PTS) platform for the detection of *E. coli* O157:H7 [74]. The design incorporated a hybridization chain reaction (HCR) amplification mechanism. Magnetic beads (MBs) functionalized with a single-stranded capture probe hybridized with an aptamer (AP) specific to *E. coli.* Upon target binding, the aptamer was released, allowing the CP strands to trigger HCR using biotin-labeled DNA polymers. Streptavidin-modified MOFs encapsulating human chorionic gonadotropin (hCG) were subsequently recruited to the polymers. The hCG acted as a signal generator on the commercial PTS, allowing visual detection with a LOD of 530 CFU/mL.

In another study focused on respiratory pathogens, a dual-channel electrochemical immunosensor was constructed using a nanocomposite of graphene oxide (GO) and Cu–MOFs for the simultaneous detection of *Mycoplasma pneumoniae* and *Legionella pneumophila* [75]. The platform employed pyrene linkers for antibody immobilization via 1-ethyl-3-(3-dimethylaminopropyl)carbodiimide–N-hydroxysuccinimide chemistry and exhibited a broad dynamic range (1 pg/mL to 100 ng/mL). The dual-sensor format was successfully validated in spiked water samples, emphasizing its real-world applicability.

The detection of foodborne pathogens in dairy matrices has also advanced significantly. A polydopamine/CoFe-MOFs@Nafion-based electrochemical immunosensor was developed for *Salmonella* detection in milk [76]. CoFe-MOFs provided structural stability and high antibody immobilization efficiency, while Nafion prevented detachment from the electrode. Electrodeposited polydopamine facilitated covalent bonding with anti-*Salmonella* antibodies via Michael addition and Schiff base reactions. The sensor exhibited a detection range of 1.38 × 10^2^ to 1.38 × 10^8^ CFU/mL, with an LOD of 1.38 × 10^2^ CFU/mL.

A similar label-free electrochemical immunosensor was fabricated by incorporating CoFe-MOFs with multi-walled carbon nanotubes (MWCNTs), followed by gold nanoparticle (AuNP) electrodeposition [77]. This hybrid enhanced conductivity and surface area for antibody anchoring, yielding a detection range of 1.04 × 10^4^ to 1.04 × 10^8^ CFU/mL and an LOD of 2.89 × 10^3^ CFU/mL.

#### 3.1.3. Dual-Mode and Multiplex Biosensing Platforms

Beyond electrochemical platforms, dual-mode optical biosensors have been developed to improve accuracy and redundancy. A colorimetric/fluorescent biosensor for *S. typhimurium* utilized magnetic covalent organic frameworks (MCOFs), gold nanoparticles, and aggregation-induced emission luminogens (AIEgens) [78]. The aptamer-mediated system prevented nanoparticle–MCOF aggregation in the presence of target bacteria, altering the signal in both detection modes. The biosensor achieved LODs of 1000 CFU/mL (colorimetry) and 10 CFU/mL (fluorescence), with smartphone-based linear discriminant analysis enhancing field usability.

**Figure 4:**
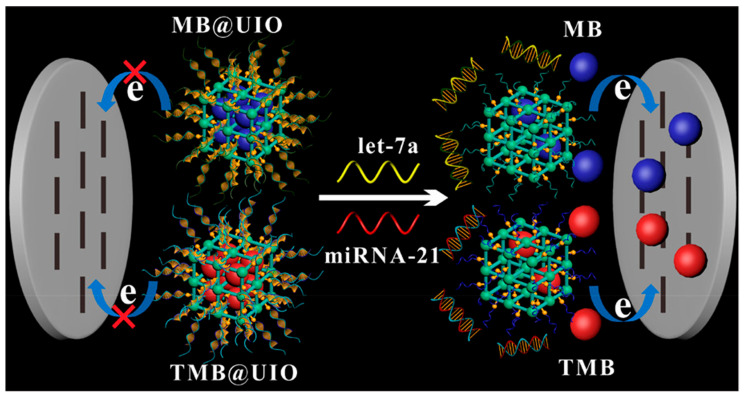
Schematic illustration of a homogeneous electrochemical biosensing platform based on functionalized UiO-66-NH_2_ MOFs for simultaneous detection of multiple microRNAs (let-7a and miRNA-21). Electroactive dyes (MB and TMB) are encapsulated within the MOFs and gated by duplex DNA. Copyright 2019 ACS [81].

A dual-mode Cu-MOF-based aptasensor targeting C-reactive protein (CRP), a biomarker elevated in bacterial and viral infections (including COVID-19), combined fluorescence and colorimetric readouts [79]. The Cu-MOF acted both as a signal quencher and enzyme mimic. Ribonucleic acid (RNA) aptamer binding to CRP released the quenched signal, enabling detection with LODs of 40 pg/mL (fluorescence) and 240 pg/mL (colorimetry), outperforming conventional assays. Moreover, though not directly microbial, a fluorometric Zn-MOF biosensor was reported for human epidermal growth factor receptor 2 (HER2), a cancer biomarker demonstrating high selectivity and a low detection limit of 1.38 pM [80]. Similarly, UiO-66-NH_2_ MOFs loaded with methylene blue and 3,3′,5,5′-tetramethylbenzidine (TMB) enabled simultaneous electrochemical detection of tumor-related microRNAs (let-7a and miRNA-21) with detection limits of 3.6 fM and 8.2 fM, respectively (Figure 4) [81]. These findings reveal that MOF-based biosensors designed for oncology can be readily repurposed for pathogen-related biomarkers such as CRP and miRNA.

Finally, a study utilizing porphyrinic covalent organic frameworks (p-COFs) though not strictly a MOF demonstrated promising photoelectrochemical detection of CRP using silver nanoparticle enhanced aptamer platforms [82]. The p-COF system showed reduced electron transfer upon CRP binding, resulting in photocurrent suppression. The material’s high photostability and conductivity provide insights into the broader applicability of crystalline porous frameworks for microbial biosensing.

### 3.2. Detection Platforms (Label-Free vs. Labeled)

MOFs are highly versatile scaffolds capable of supporting diverse detection strategies [83]. In biosensing, MOFs can be tailored to operate through either label-free or labeled detection platforms, depending on whether external signal reporters are used. Label-free platforms offer simplicity and faster readouts by directly converting target recognition events into measurable signals, whereas labeled platforms employ fluorescent tags, enzymes, nanozymes, or amplification reactions to enhance signal sensitivity and specificity [84,85,86].

#### 3.2.1. Label-Free Detection Platforms

Label-free biosensors are increasingly favored in point-of-care diagnostics and real-time monitoring due to their inherent simplicity, cost-effectiveness, and minimal sample preparation requirements. These platforms exploit direct interactions between the MOF sensor surface and the target pathogen, which result in detectable optical or electrochemical changes without the need for additional signal labels.

In the electrochemical arena, MOFs have proven to be robust platforms for nucleic acid sensing applications. A polyaniline@nickel-MOF (Ni-MOF) nanocomposite was developed as a highly sensitive, label-free biosensor for detecting hepatitis C virus (HCV) RNA without the need for amplification [87]. The nanocomposite was assembled layer by layer onto a glassy carbon electrode functionalized with a DNA probe. Hybridization with target RNA altered the electrochemical impedance, allowing for detection across an exceptionally wide concentration range (1 fM to 100 nM) with a detection limit as low as 0.75 fM. This performance rivals the sensitivity of RT-PCR, illustrating the power of MOF-based materials in nucleic acid diagnostics.

Similarly, MOFs have shown strong promise for exosome detection, which is relevant in both infectious and non-infectious diseases. A label-free biosensor based on Zr-MOFs encapsulating methylene blue was employed for the detection of exosomes from glioblastoma multiforme (GBM) [88]. By utilizing a peptide that binds to epidermal growth factor receptor markers overexpressed in GBM exosomes the MOF–electrode interface can directly transduce the binding events into electrochemical signals. The biosensor achieved a detection limit of 7.83 × 10^3^ particles/μL with a wide detection range (9.5 × 10^3^ to 1.9 × 10^7^ particles/μL), offering a non-invasive diagnostic tool for early disease detection.

**Figure 5:**
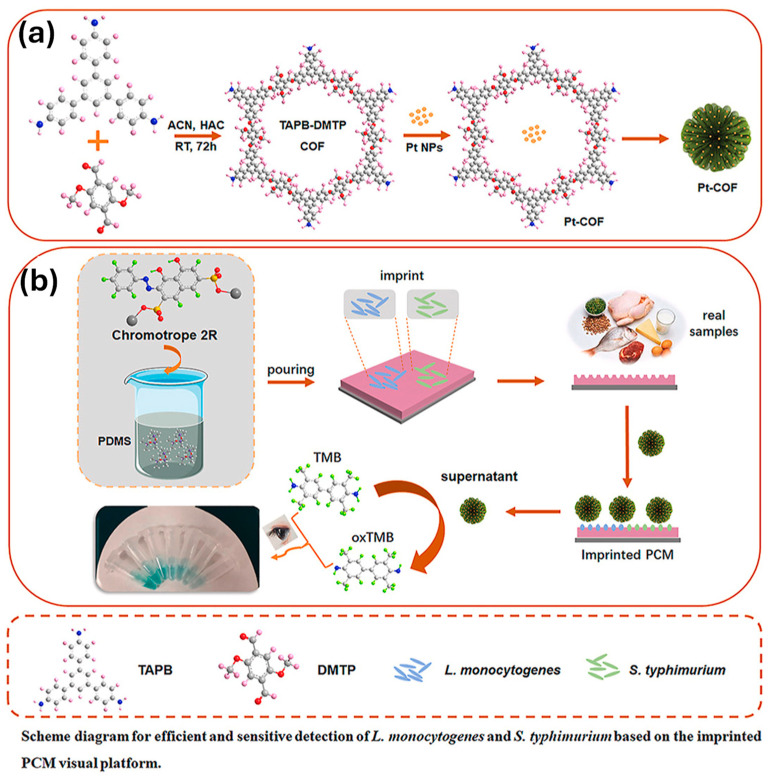
(**a**) The synthesis procedure of the Pt-covalent organic framework (Pt-COF). (**b**) Illustration of the imprinted PCM visual platform used for the rapid and sensitive detection of *L. monocytogenes* and *S. typhimurium*. Copyright 2024 Elsevier [89].

Colorimetric detection systems have benefited from MOF-based innovations. For instance, a visual dual-pathogen detection platform was developed using a positively charged Pt-COF nanozyme and a Chromotrope 2R–modified membrane to simultaneously identify *L. monocytogenes* and *S. typhimurium* (Figure 5) [89]. The positively charged nanozyme facilitated electrostatic adsorption onto the pathogen-enriched membrane, enabling subsequent catalytic oxidation of TMB in the presence of H_2_O_2_. The assay achieved dual detection limits of 1.31 and 1.61 CFU/mL, demonstrating the potential of label-free MOF nanozymes for multiplexed and field-deployable detection.

In another application tailored for food safety, CoFe-MOFs hybridized with graphene were employed for label-free electrochemical detection of *Salmonella* in milk [90]. The incorporation of Au–NH_2_ not only stabilized the antibody immobilization but also enhanced the conductivity and surface area. The resulting sensor displayed an impressive linear range of 2.4 × 10^2^ to 2.4 × 10^8^ CFU/mL, with an LOD of 1.2 × 10^2^ CFU/mL, confirming its suitability for complex sample matrices.

Further optical biosensing research led to, a platform based on ultrathin MOF nanosheets (MOF-NSs) was designed for label-free, multiplexed DNA detection [91]. These nanosheets acted as highly selective adsorbents for dye-labeled ssDNA probes, which were released upon hybridization with target DNA from various pathogens. This release restored fluorescence, enabling simultaneous detection of *S. enterica*, *L. monocytogenes*, and Vibrio parahaemolyticus, with detection limits as low as 15 pM. Taken together, these label-free approaches underscore the utility of MOFs in simplifying biosensor design while maintaining high sensitivity and specificity. Their adaptability across optical and electrochemical modalities makes them ideal candidates for rapid, on-site pathogen detection.

#### 3.2.2. Labeled Detection Platforms

While label-free systems emphasize simplicity, labeled biosensors are designed to maximize signal output through the strategic incorporation of external signal reporters. These platforms often yield superior sensitivity and specificity, and are particularly valuable in early-stage diagnostics where pathogen levels are extremely low.

One of the most compelling examples is a microfluidic immunosensor for *Salmonella* detection, which integrated Fe-MIL-88NH_2_ MOF nanocubes loaded with platinum nanoparticles (PtNPs) as peroxidase-mimicking nanozymes [92]. The sensor used a sandwich immunoassay format where magnetic nanoparticles first captured the target bacteria, and the MOF-PtNP probes catalyzed H_2_O_2_ decomposition to generate oxygen. Oxygen was detected using a smartphone-based thermal imaging system, enabling visual, label-based detection with a sensitivity of 93 CFU/mL within one hour. This example demonstrates the coupling of labeled MOF platforms with portable readouts to enable the real-time detection of pathogen.

Further extending this approach, a Cu-MOF/poly(3,4-ethylenedioxythiophene):poly(styrenesulfonate) (PEDOT:PSS) composite platform was developed for the labeled electrochemical detection of *E. coli* O157:H7 [93]. Sulfonic acid groups were introduced into the MOF to facilitate antibody attachment, and the antigen–antibody interaction was transduced via enhanced electron transfer in the conductive composite. This system achieved a wide detection range (3 × 10^2^–3 × 10^8^ CFU/mL) with a detection limit of 7.4 CFU/mL, illustrating how labeled strategies can be adapted for robust electrochemical biosensing.

Visual biosensors have also benefitted from enzyme encapsulation in MOFs. A prominent example involved enzyme-loaded ZIF-8 particles for the detection of *E. coli* O157:H7 [94]. The stable, porous MOF matrix preserved enzyme activity, enabling colorimetric detection with a detection limit of just 1 CFU/mL, and visible detection by eye down to 10 CFU/mL in complex matrices such as milk, seawater, and cosmetics. This approach exemplifies how MOFs can enhance enzyme stability while allowing simple and effective visual detection.

Despite the sensitivity advantages of enzymatic labels, immobilization often reduces their catalytic efficiency. This limitation was addressed using MOF-encapsulated iron porphyrin nanozymes, such as MILL-88@TcP [95]. Upon ethanol mediated release, the tetraphenylporphyrin (TcP) exhibited 1430-fold higher catalytic efficiency compared to traditional MIL-88. This nanozyme-linked immunosorbent assay format enabled sensitive detection of *S. typhimurium* with a detection limit of 1.68 × 10^2^ CFU/mL, which is over 500 times more sensitive than conventional horseradish peroxidase (HRP)-based ELISAs while maintaining excellent specificity.

Incorporating nucleic acid amplification into MOF-based labeled systems can further enhance their sensitivity. One example is a fluorescence biosensor combining recombinase-aided amplification (RAA), CRISPR/Cas12a-based recognition, and ZIF-8 loaded with fluorescein sodium [96]. In this system, ZIF-8 served both as a carrier and a pH-sensitive release vehicle for FLS. Upon target recognition, the MOF released the dye, producing intense fluorescence for sensitive detection of *S. typhimurium* with a detection limit of 1.3 × 10^2^ CFU/mL within two hours.

Other innovative methods include dual-aptamer sandwich immunoassays and magnetic separation. A colorimetric sensor for *E. coli* uses streptavidin-labeled Cu-MOFs as catalytic probes with peroxidase-like activity [97]. This system achieved a detection limit of 2 CFU/mL and a linear range from 16 to 1.6 × 10^6^ CFU/mL. Similarly, a magnetic separation-enhanced colorimetric platform was developed using platinum-loaded ZIF-8 (Pt@ZIF-8) nanozymes for detecting *S. typhimurium*, reaching a detection limit of 11 CFU/mL in poultry samples [98]. This biosensor achieved a low detection limit of 11 CFU/mL, with a linear range of 10^1^–10^4^ CFU/mL, and demonstrated high recovery (~109.8%) in spiked chicken carcass samples. The use of MOF-based nanozymes in a labeled configuration, combined with efficient magnetic separation, highlights the potential of this platform for sensitive and scalable foodborne pathogen detection without the need for pre-enrichment steps.

For rapid POCT, a pipette tip-based biosensor was devised, incorporating immune-functionalized nickel meshes and fluorescent MOFs modified with boronic acid groups (MOFs@B(OH)_2_) [99]. The platform enabled the selective binding of *Salmonella* in a conical separation unit, followed by an acid-triggered release of the fluorescent label. The detection limit reached 18.8 CFU/mL, and results were available in just 20 min, highlighting the translation of labeled MOF biosensors into practical diagnostic tools. Moreover, a labeled electrochemical immunosensor was developed for the sensitive and rapid detection of *S. typhimurium* in milk. The sensor incorporated platinum nanoparticles and a Co/Zn-MOF supported on carboxylated MWCNTs, which synergistically improved both the sensitivity and signal stability [100]. In the optimized system, antibodies specific to *S. typhimurium* were immobilized on the sensor, enabling selective recognition and signal generation. The immunosensor exhibited a wide linear detection range from 1.3 × 10^2^ to 1.3 × 10^8^ CFU/mL and a low detection limit of 94 CFU/mL. The platform also demonstrated strong specificity, reproducibility, and stability, showing strong promise for extension to other labeled detection strategies targeting foodborne pathogens.

Overall, labeled MOF-based detection platforms offer amplified signals and higher sensitivities than their label-free counterparts. These systems can be tailored for specific readouts (fluorescence, electrochemical, and colorimetric) and integrated with microfluidics or portable devices, making them powerful tools for sensitive, real-world biosensing applications.

### 3.3. Performance Metrics (Sensitivity, Selectivity, LOD)

**Figure 6:**
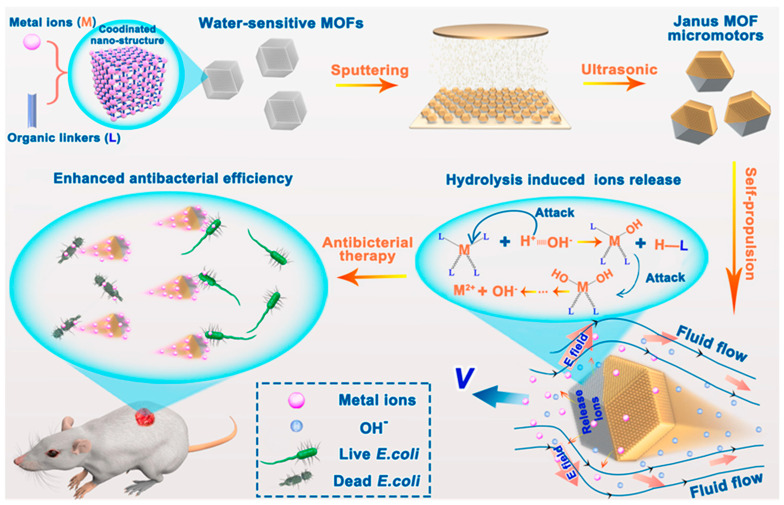
Schematic representation of the fabrication of MOF-based micromotors and their application in antibacterial wound treatment. Copyright 2022 ACS [101].

The performance of MOF-based optical biosensors is primarily evaluated in terms of sensitivity, selectivity, and LOD, all of which are critical for effective pathogen diagnostics. Their high surface area and adjustable pore structures enable MOFs to achieve low detection limits by increasing analyte capture and signal response. The performance of biosensors and therapeutic platforms is often gauged by their sensitivity, selectivity, and functional efficiency. In a recent study, self-fueled MOF-based micromotors were designed to combine autonomous motion with intrinsic antibacterial functionality, offering a highly integrated sensing-therapeutic approach. These micromotors spontaneously degrade in aqueous environments, releasing ionic species that not only fuel their self-propulsion via ionic diffusiophoresis but also act as antibacterial agents. The release of metal cations enhanced the selective killing of *E. coli*, with motion-assisted activity significantly improving antibacterial efficacy (Figure 6) [101]. In an in vivo wound model, this system led to accelerated wound healing, demonstrating both biological specificity and functional efficiency. Although traditional biosensing metrics such as LOD have not been directly reported, the system’s selective antibacterial action and self-powered delivery of the system highlight its potential for future high-performance biosensing and theranostic applications, particularly where both biological targeting and therapeutic impact are required. A comprehensive summary of MOF-based biosensors, including sensor materials, target microorganisms, detection methods, detection limits, and linear ranges, is provided in Table 2.

Most MOF-based biosensors seem to favor optical detection, likely because it is fast, often does not require labels, and can be adapted for portable or even wearable devices. One practical perk is that some of these systems allow for visual readouts or smartphone-based monitoring, which is handy in field settings. Electrochemical sensors, on the other hand, can achieve extremely low detection limits and precise quantification, but they often come with more demanding instrumentation and careful electrode design. Each method has its quirks: optical sensors may struggle with photobleaching or interference from complex samples, while electrochemical setups can be tricky to scale for widespread deployment. In the end, the choice often depends on the target analyte, how sensitive the measurement needs to be, and the conditions in which the sensor will be used, suggesting there is no one-size-fits-all solution.

## 4. MOF-Based Optical Biosensors for Environmental Monitoring

MOF-based optical biosensors are promising tools for environmental monitoring due to their exceptional sensitivity, tunability, adaptability, and ease of use. These materials offer unique advantages stemming from their highly porous architectures, large surface areas, and flexible chemical functionalities, which enable selective interaction with a wide range of environmental analytes to be achieved. MOFs can be functionalized with luminescent ligands, chromophores, enzyme mimics, or responsive molecules to produce measurable optical responses such as fluorescence, colorimetric changes, and photoluminescence upon interaction with specific contaminants. The integration of these responsive MOFs into platforms such as fiber optics, paper chips, or hybrid nanocomposites further enhances their usability in portable, field deployable formats. This makes MOF-based optical biosensors especially suitable for real-time, on-site analysis of environmental pollutants including heavy metals, pesticides, toxins, and microbial agents [102,103].

The accumulation of toxic heavy metals in the environment, particularly in water sources, poses a significant threat to public health and has been linked to neurodegenerative diseases like Alzheimer’s [104]. Optical biosensors offer a powerful tool for environmental monitoring by enabling the rapid, selective, and ultra-sensitive detection of trace levels of heavy metals such as mercury, cadmium, lead, and arsenic, thereby supporting early warning and contamination control efforts [105].

### 4.1. Detection of Pollutants (Heavy Metals, Pesticides, Toxins, etc.)

MOF-based optical biosensors enable the rapid and sensitive detection of environmental pollutants such as heavy metals, pesticides, and toxins by translating molecular interactions into fluorescence or colorimetric signals [106,107]. Their customizable pore structures and surface chemistry allow for selective recognition, but performance in complex natural matrices can deviate from laboratory benchmarks.

For heavy metal detection, zirconium-based dMOR-2, functionalized with 2-picolylamine ligands, simultaneously served as an optical sensor and adsorbent for Cu^2+^, Pb^2+^, and Hg^2+^, achieving detection limits below 2 ppb in aqueous systems [108]. Dispersed in water with polyvinylpyrrolidone, dMOR-2 demonstrated outstanding fluorescence sensing capabilities for Cu^2+^, Pb^2+^, and Hg^2+^ with detection limits below two parts per billion (ppb), even in complex aqueous matrices. Its sensing performance was attributed to chelation-induced fluorescence modulation by the picolylamine group. In parallel, dMOR-2 was also integrated into a calcium alginate composite and exhibited high adsorption capacities for these metal ions, thereby highlighting its multifunctional role in detection and remediation. While effective in spiked samples, natural waters with high organic content or turbidity may attenuate fluorescence signals, necessitating pretreatment or anti-fouling strategies.

Moreover, Pu et al. reported an azobenzene-based luminescent probes were incorporated into a bio-MOF-1 matrix to create optical biosensors for the detection and adsorption of heavy metal ions, particularly Cu^2+^ [109]. These MOF-integrated sensors (P1@BMOF and P2@BMOF) exhibited high selectivity, low detection limits (as low as 0.20 μM), and enhanced stability, demonstrating effective fluorescence quenching responses and strong adsorption capacities, making them promising tools for environmental monitoring of toxic metal pollutants. A different approach involving defect engineering was employed to enhance the sensitivity of lanthanide-based luminescent MOFs. Specifically, Eu@UiO-MOFs were synthesized with modulator-induced missing-linker defects, which fine-tuned the photophysical properties of the framework [110]. The defect-rich Eu@UiO-MOFs, with an average of 0.53 missing linkers per Zr_6_ node, exhibited significantly amplified fluorescence responses upon Cd^2+^ binding and enabled ultrasensitive detection at a limit of 114 ppb. These results emphasized the importance of structural defect modulation in improving the performance of MOF-based optical biosensors.

For chromium detection, a fiber-optic sensor platform was developed by coating a U-bent fiber optic probe with ZIF-67 via in situ deposition and subsequent thermal treatment [111]. The fabricated fiber optic sensor/ZIF-67 sensor took advantage of the evanescent wave absorbance at 395 nm, which is a characteristic of Cr(VI), and achieve a detection limit as low as 1 ppb. In addition to its wide linear detection range and rapid response, the sensor displayed high selectivity, operational stability in real water samples, and robust shelf life making it highly suitable for practical deployment in environmental monitoring. In another example, a manganese-based MOF (SM-1), formed from 2,5-furandicarboxylic acid and 4,7-phenanthroline, exhibited anionic framework characteristics with uncoordinated nitrogen sites that enhanced its stability and analyte interaction [112]. SM-1 functioned as a dual-mode fluorescent sensor, showing a “turn-on” response for Ag^+^ and Cd^2+^, and a “turn-off” signal for Hg^2+^ ions. The MOF achieved low detection limits in aqueous media and demonstrated potential for environmental remediation through reversible iodine adsorption.

To address the detection of spectroscopically silent ions, an optical biosensor targeting Pb^2+^ was designed by growing MOF-5 in situ on tannic-acid-functionalized gold nanoparticles deposited on a U-bent fiber [113]. This plasmonic absorption-based sensor translated the refractive index shift upon Pb^2+^ binding into enhanced plasmonic signals, achieving a detection limit of 0.5 ppb. The sensor exhibited outstanding selectivity, stability, and operational effectiveness for complex sewage samples, demonstrating its suitability for on-site water quality surveillance.

Furthermore, a fluorescence “turn-off” immunosensor was developed using amine-functionalized MIL-101(Fe) MOFs conjugated with monoclonal antibodies specific to Pb^2+^ ions [114]. The biosensor exhibited high aqueous stability and biocompatibility, enabling the selective detection of Pb^2+^ at concentrations as low as 9.51 ppb in real water samples, meeting the safety limits set by the WHO and EPA. Complementing these optical platforms, a dual-mode biosensor combining photoelectrochemical and electrochemical (EC) responses was constructed using a phthalocyanine-based covalent organic framework (CoPc-PT-COF) covalently grown on a Cu-MOF substrate [115]. This hybrid structure combines the high photo- and electroactivity of phthalocyanine and bipyridine units with the porous and functional surface of the MOF. The enhanced photoelectric properties of CoPc-PT-COF@Cu-MOF, along with its favorable band structure, enabled an amplified signal output in both PEC and EC detection modes. DNA probes immobilized on the heterostructure selectively recognized Cr^3+^ ions through specific interactions, allowing for highly sensitive detection in aqueous environments. The biosensor achieved markedly low detection limits of 14.5 fM (PEC) and 22.9 fM (EC) across a wide concentration range (0.1 pM to 100 nM), with excellent selectivity, stability, and reproducibility. It also successfully detected trace amounts of Cr^3+^ in real-world samples such as river and tap water. This work highlights the potential of MOF-based photoelectrochemical biosensors for advanced environmental monitoring, particularly for tracking ultra-trace levels of toxic heavy metals in complex water matrices.

**Figure 7:**
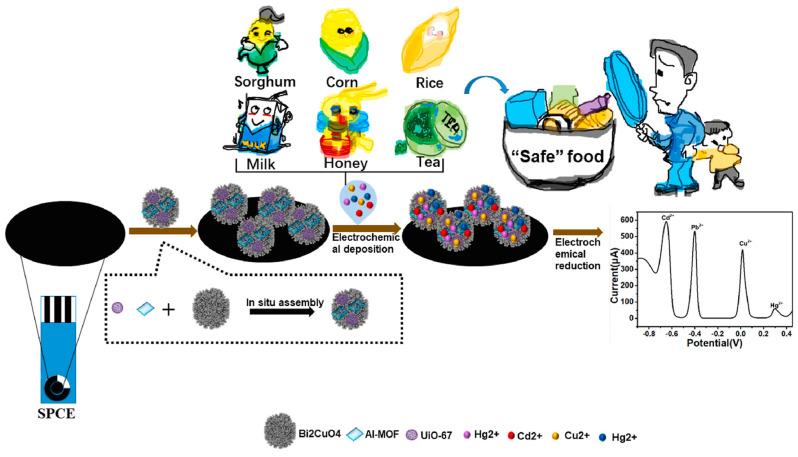
Illustration of the Bi_2_CuO_4_@Al-MOF@UiO-67/SPCE sensor used for detecting ${Cd}^{2+}$, ${Cu}^{2+}{Pb}^{2+}$, and ${Hg}^{2+}$ ions in food samples. Copyright 2024 Elsevier [116].

Though not directly based on optical readout, a hybrid electrochemical sensor combining Bi_2_CuO_4_, UiO-67, and Al-MOF achieved simultaneous detection of Cd^2+^, Cu^2+^, Pb^2+^, and Hg^2+^ with remarkable detection limits down to the picomolar range (e.g., 0.02 pM for Cd^2+^) [116]. This unique architecture not only provides superior electrocatalytic redox activity but also ensures high stability, reproducibility, and selectivity (Figure 7). Remarkably low detection limits were achieved for each metal ion, down to picomolar levels (e.g., 0.02 pM for Cd^2+^). The detection mechanism involves both adsorption and electrochemical accumulation of metal ions on the MOF-modified electrode surface. To demonstrate real-world applicability, the sensor was successfully used to detect heavy metals in various complex food matrices such as rice, milk, and tea, highlighting its potential for environmental and food safety monitoring.

### 4.2. Detection of Pesticides and Herbicides

The pervasive use of pesticides and herbicides in agriculture introduces toxic residues into ecosystems, necessitating the development of robust detection methods. MOF-based optical biosensors offer field-friendly alternatives to chromatographic techniques because of their rapid response and visual readouts.

One such example is a Cu-MOF nanoprobe developed using a one-pot reaction between Cu^2+^, 5-aminoisophthalic acid (AIA), and trimesic acid [117]. The MOF exhibited strong quenching behavior due to ligand-to-metal charge transfer and PET. Upon glyphosate exposure, competitive binding disrupted the framework, releasing the AIA ligand and triggering fluorescence recovery. The sensor achieved a detection limit as low as 33 nM, which is significantly lower than the U.S. EPA maximum contaminant level. In addition to environmental sensing, the probe enabled real-time imaging of glyphosate in plant tissues and the screening of agricultural samples.

Another effective platform is a paper-based biosensor composed of acetylcholinesterase (AChE) immobilized on a ZIF-8-modified cellulose matrix [118]. The MOF encapsulation enhanced enzyme stability under variable pH, temperature, and storage conditions, while the peroxidase-like activity of acetylthiocholine iodide allowed for efficient optical signal generation. The sensor achieved a low detection limit of 0.29 μg/L and a linear range of 0.5–50 μg/L for dichlorvos within 20 min. The integration with a 3D-printed holder and smartphone readout enabled portable, instrument-free POCT. This study demonstrates the potential of MOF-enabled colorimetric biosensors as reliable, field-deployable tools for environmental and agricultural monitoring, particularly for ensuring food safety and pesticide regulation.

### 4.3. Detection of Microbial Pathogens

MOF-based optical biosensors have also shown considerable progress in the detection of pathogenic microorganisms, offering a noninvasive and highly specific alternative to culture-based methods. By integrating biological recognition elements such as antibodies and aptamers, MOFs can detect bacteria through fluorescence quenching or enzymatic signal recovery. A notable development in this domain is a terbium-based MOF constructed with 1,3,5-benzenetricarboxylic acid (Tb-BTC), functionalized with anti-*E. coli* antibodies [119] to enable selective bio-recognition. The optical response, based on changes in fluorescence intensity, allows for the detection of *E. coli* across a wide concentration range (1.3 × 10^2^ to 1.3 × 10^8^ CFU/mL), with an exceptionally low detection limit of 3 CFU/mL and a rapid response time of just 5 min. This work highlights the significant potential of MOF-based fluorescent platforms for optical biosensing applications. Owing to its high sensitivity, selectivity, fast response, and successful demonstration in complex matrices such as fruit juice, the Tb-BTC system shows promise for the real-time environmental monitoring of microbial contamination in water and food sources.

Similarly, a zirconium–praseodymium-based MOF nanozyme (ZrPr-MOF) was combined with aptamer-based recognition to develop a colorimetric sensor for *S. typhimurium* [120]. The presence of *S. typhimurium* disrupts the aptamer–nanozyme interaction, restoring catalytic activity and enhancing the colorimetric response. The biosensor showed excellent sensitivity under optimized conditions, with a linear detection range of 10^2^–10^8^ CFU/mL and a low detection limit of 37 CFU/mL. Additionally, a paper-based format of the biosensor was demonstrated for portable, point-of-care detection, successfully identification of *S. typhimurium* in spiked milk samples. This study highlights the potential of MOF-based nanozyme optical biosensors for rapid, selective, and on-site microbial monitoring in food safety and environmental surveillance applications.

### 4.4. Detection of Biochemical and Volatile Environmental Contaminants

In addition to heavy metals, pesticides, and pathogens, environmental pollutants encompass a broad category of volatile and biochemical substances, including quorum sensing molecules, plant stress markers, VOCs, biochemical waste, and gaseous pollutants. MOFs, owing to their high porosity, structural tunability, and optical versatility, have been successfully employed to detect these diverse targets with excellent sensitivity and selectivity using a variety of optical modalities.

For instance, quorum sensing molecules such as N-(3-oxodecanoyl)-L-homoserine lactone (3-O-C10-HL), which mediate microbial communication and contribute to biofilm formation, have been detected using a sophisticated optoelectronic biosensor system [121]. This system integrates a Z-scheme heterojunction comprising CAU-17 MOF and Bi_2_S_3_ nanostructures, configured into an organic photoelectrochemical transistor. Functionalized with self-screening aptamers, the sensor enables highly selective detection of 3-O-C10-HL down to 0.441 pM, presenting a promising platform for marine biofouling monitoring and early-stage surveillance of microbial activity.

**Figure 8:**
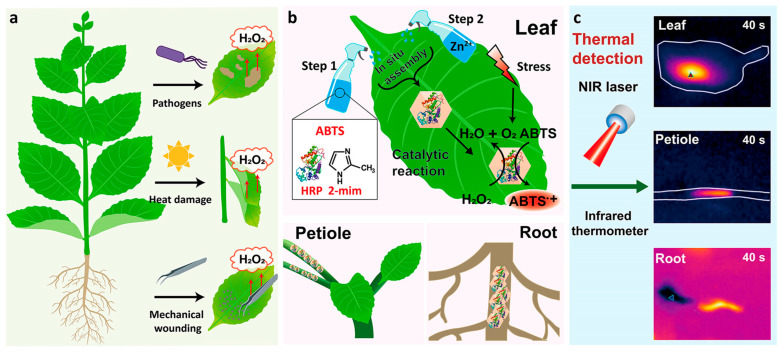
Illustration of ZIF-8-based plant H_2_O_2_ biosensor. (**a**) Nicotiana benthamiana under stress (e.g., pathogens, heat, wounding) produces H_2_O_2_. (**b**) A mixture of HRP, ABTS, and 2-methylimidazole is sprayed onto plant tissues, followed by Zn^2+^ to form ZIF-8 encapsulating the sensing components in situ. (**c**) H_2_O_2_ oxidizes ABTS to ABTS•^+^, which generates a thermal signal under NIR light, enabling remote detection via a thermometer [122].

In the realm of plant health diagnostics, hydrogen peroxide (H_2_O_2_) serves as a key signaling molecule that indicates biotic and abiotic stress. Traditional methods for H_2_O_2_ detection typically involve destructive sample processing, which can further stress the plant and are labor-intensive. To overcome these limitations, the authors developed a ZIF-8-based color-to-thermal biosensor formed directly on plant tissues (roots, petioles, and leaves) by spraying a mixture of HRP, 2,2′-Azino-bis(3-ethylbenzothiazoline-6-sulfonic acid) (ABTS), and ZIF-8 precursors [122]. Upon exposure to near-infrared (NIR) light, the oxidized product (ABTS•^+^) produces heat, allowing thermal signal detection of sub-micromolar concentrations of H_2_O_2_ (Figure 8). Plant tissues have minimal absorption in the NIR region; therefore, the biosensor enables remote optical readout with a high signal-to-noise ratio, without damaging the plant. This study demonstrates the utility of MOF-based optical biosensing platforms for noninvasive, real-time monitoring of plant stress responses, offering significant promise for applications in precision agriculture, environmental monitoring, and plant physiology research.

VOCs, including industrial solvents such as ethanol, acetone, and methanol, are significant air pollutants due to their toxicity and flammability. An LSPR-based optical fiber sensor was developed using a layer-by-layer deposition of gold nanoparticles and HKUST-1 on a fiber tip [123]. Varying the number of MOF coating cycles (40, 80, and 120) allowed for the tuning of the sensitivity of the sensor, with significant optical responses observed only at higher coating thicknesses. The sensor exhibited a measurable redshift in resonance wavelength upon VOC exposure due to refractive index changes from VOC adsorption into the MOF layer, with high sensitivity and low detection limits for acetone, ethanol, and methanol. The sensor also demonstrated reversible behavior, rapid response and recovery times, and minimal interference from humidity and temperature, highlighting its promise as a reliable MOF-based optical platform for real-time environmental VOC monitoring.

In addition to air and plant stress analytes, MOF-based biosensors have been used to detect biochemical pollutants such as urea and morphine. A tapered single mode coreless single-mode optical fiber, functionalized with urease-loaded ZIF-8 facilitates, allowed urea detection via enzyme-mediated changes in local refractive index [124]. The detection mechanism relies on the binding of urea to urease, which induces changes in the local refractive index, leading to measurable resonance wavelength shifts in the fiber’s transmission spectrum. The biosensor demonstrated a linear optical response to urea concentrations ranging from 1 to 10 mM, with a detection limit of 0.1 mM and sensitivity of 0.8 mM/RIU when operated over a broadband light source (1525–1590 nm). Performance evaluation using real samples confirmed the sensor’s high selectivity and sensitivity, establishing its suitability for optical biosensing applications. This work underscores the potential of MOF-enzyme hybrid systems, particularly ZIF-8/urease, for the environmental monitoring of biochemical contaminants, such as urea in water, offering a robust and precise sensing platform for real-world applications.

Complementing this approach, a hydrogel-confined dual-enzyme cascade system urease–Fe-cdDNA dual-enzyme cascade(UFD-DEC), comprising Fe-cdDNA nanozymes and urease, produced a visible color change upon urea decomposition [125]. By precisely tuning the catalytic activity of Fe-cdDNA nanozymes using DNA control, the cascade reaction is significantly accelerated while maintaining high structural stability. Encapsulated within a hydrogel matrix, the system forms a portable “lab-in-a-tube” device capable of on-site urea detection. The readout is facilitated via a smartphone-based image analysis algorithm, which enabled real-time, colorimetric signal interpretation with a detection limit of 0.12 mmol/L. This work showcases a customizable, low-cost, and miniaturized biosensor platform with strong potential for point-of-care diagnostics and environmental monitoring, particularly for urea profiling in agricultural or clinical contexts.

Wearable and physiological biosensing was also demonstrated using a MOF-based colorimetric sensor for transcutaneous CO_2_ detection [126]. It offers high sensitivity and selectivity, detecting CO_2_ levels as low as 26 ppm across a wide range (0–2% CO_2_) while resisting interference from skin gases. Human trials have confirmed its clinical potential, and its compact and responsive design suits its applications in sports and exercise for respiratory monitoring. Overall, this MOF-complementary metal–oxide–semiconductor (CMOS) sensor’s affordability, reversibility, and sensitivity make it ideal for wearable remote health tracking devices.

**Figure 9:**
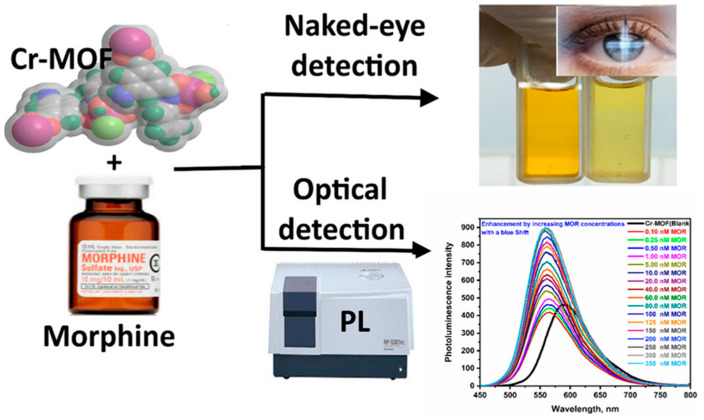
Illustration of the MOF assembly and applications for optical based biosensing. Copyright 2020 ACS [127].

Additionally, a Cr(III)-based MOF nanoparticle fluorescent sensor was engineered for the optical detection of morphine (Figure 9) [127]. The Cr(III)-MOF-NPs exhibited a characteristic emission at 593 nm, which shifted to 566 nm with enhanced intensity upon the addition of MOR, accompanied by a visible color change from brown to yellow, enabling rapid and selective optical detection. The developed PL sensor showed high sensitivity with a detection limit of 0.167 nM and strong selectivity for MOR across a concentration range of 0.1 to 350 nM. It was successfully applied to ultrasensitive morphine quantification in spiked serum and urine samples, demonstrating its practical utility in complex biological matrices.

Additionally, enhancing plasmonic optical biosensors with transition metal carbide (MXene)-MOF hybrids has improved the sensitivity for biochemical detection [128]. Furthermore, combining Ti_3_C_2_ with a terbium-based MOF (Tb-BTC) enabled the fabrication of a selective optical biosensor for hemoglobin. The Tb-BTC MOF selectively binds to the iron center of hemoglobin, thereby enhancing the specificity of the sensor. The sensor demonstrated reliable detection of hemoglobin in the range of 100 to 500 μg/mL and maintained selectivity even in the presence of interfering biomolecules such as immunoglobulin, glucose, uric acid, and bovine serum albumin. This hybrid MXene-MOF platform shows significant potential for sensitive and selective optical biosensing in environmental and clinical monitoring contexts. A summary of the key MOF-based optical biosensors discussed in this section, including their materials, target analytes, detection methods, performance metrics, and references, is provided in Table 3 for easy comparison and overview.

## 5. Integration with Advanced Technologies

The integration of MOF-based optical biosensors with advanced technologies such as microfluidics, portable electronics, artificial intelligence (AI), and the Internet of Things (IoT) is driving a new generation of smart sensing platforms for real-time pathogen and environmental monitoring. These synergistic advancements have significantly expanded the practical applicability of MOF-based optical biosensors in diverse settings ranging, from clinical diagnostics to environmental safety.

### 5.1. Portable-Based Sensing

Smartphone-based sensing is a powerful platform for portable and user-friendly detection by using MOF-based optical biosensors [129]. With built-in cameras, advanced processors, and connectivity, smartphones can capture and analyze optical signals such as fluorescence or colorimetric changes generated by MOF sensors. This approach enables rapid, on-site detection of pathogens or pollutants without the need for complex instrumentation. Moreover, smartphone apps can process data in real time, display results, and upload information to cloud platforms for remote monitoring [130,131,132]. The widespread availability and versatility of smartphones make them ideal tools for expanding the accessibility and scalability of MOF-based biosensing technologies.

A smartphone-integrated electrochemical biosensing platform was developed for on-site toxicity evaluation of heavy metal ions, specifically Cd^2+^, Pb^2+^, and Hg^2+^, using a Hep G2 cell response model [133]. The system uses a reduced graphene oxide/molybdenum disulfide composite electrode for enhanced biocompatibility and signal amplification, with DPV capturing electrochemical changes linked to cytotoxicity. This compact sensor interfaces with a smartphone for data acquisition and visualization, enabling accurate real-time monitoring (IC_50_ values: Cd^2+^ = 49.83 μM, Pb^2+^ = 733.90 μM, and Hg^2+^ = 36.94 μM). Its integration supports food and environmental safety assessments, confirmed by the MTT assay, microscopy, and flow cytometry.

Another portable POCT platform has been designed for rapid detection of multiple heavy metal ions (HMIs) in milk. The system uses a disposable disk with six screen-printed electrodes (SPEs) and MOFs to amplify the electrochemical signal of methylene blue [134]. DPV enabled highly sensitive detection of Cd^2+^, Hg^2+^, Pb^2+^, and As^3+^, with detection limits as low as 0.022–0.073 ppb, all within four minutes. Successfully applied to milk samples, this flexible and efficient MOF-assisted platform offers a practical solution for on-site environmental and food safety monitoring.

Li et al. introduced a 3D-printed, smartphone-based optical sensing device as a portable, low-cost substitute for conventional ultraviolet–visible spectroscopy (UV-vis) spectrometers for detecting heavy metals [135]. This device enables sensitive and accurate measurements without the need for bulky equipment or trained personnel. Its performance was validated through the successful detection of four common heavy metal ions in real water samples, showing a strong correlation with the results from standard UV-vis instruments. This approach highlights the potential of smartphone-based sensors for on-site environmental monitoring, offering high sensitivity, ease of use, and cost-effectiveness.

A cost-effective, smartphone-based POCT system was developed for simultaneous detection of multiple metal ions, including Cd^2+^, Cu^2+^, Hg^2+^, and Pb^2+^ [136]. It integrates custom electrodes with a lightweight handheld electrochemical analyzer, allowing real-time data acquisition via a dedicated mobile app (DHMI). DPV enabled sensitive, broad-range detection, and the system demonstrated performance comparable to inductively coupled plasma mass spectrometry in real water samples, offering a user-friendly and reliable tool for environmental surveillance.

A smartphone-integrated chemiresistive sensor was engineered for label-free detection of Pb^2+^ in water. Using micro-interdigitated electrodes functionalized with GO and β-cyclodextrin, the device achieved a detection limit as low as 100 pM [137]. The readout system was compact and energy-efficient, and the integration of machine learning (ML) enhanced the classification accuracy in complex matrices.

**Figure 10:**
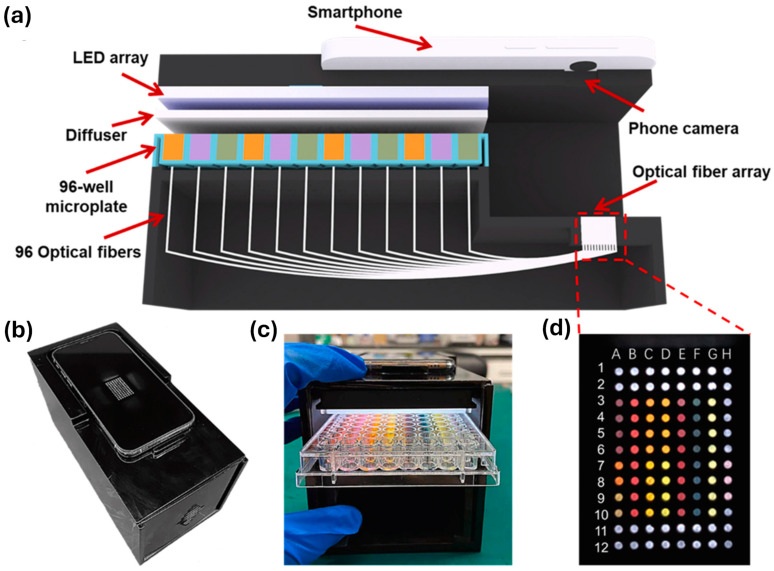
Overview of the compact smartphone-based CSA analysis system. (**a**) Diagram showing the device setup, which includes a smartphone, LED array, light diffuser, optical fiber bundle, and the CSA component. (**b**) Real-life image of the complete device. (**c**) Close-up view of the device’s front panel. (**d**) Captured image of the CSA, reduced in scale, using the portable platform. Copyright 2022 Elsevier [138].

Another portable smartphone-based colorimetric sensing platform was developed to detect heavy metal ion pollution in industrial wastewater. The sensor array used plasmonic nanocolorants and chromophores to detect 13 HMIs (e.g., Hg^2+^, Cd^2+^, Pb^2+^, and Cr^6+^) at ppm levels within 15 s [138]. Optical fibers transmit sensor images to the smartphone’s CMOS imager, generating distinct RGB fingerprints analyzed via multivariate pattern recognition techniques (Figure 10). The system effectively identifies both individual ions and complex wastewater sources (e.g., electroplating, battery, metallurgical, pesticide) with high reproducibility. Integrated with edge computing and IoT cloud capabilities, this smartphone-based platform offers a low-cost, rapid, and intelligent solution for environmental monitoring, water quality assessment, and pollution source tracing.

Xiao et al. developed a paper-based microarray platform with smartphone-based fluorescence detection using CDs for simultaneous detection of Hg^2+^, Pb^2+^, and Cu^2+^ in Pearl River water [139]. With no need for complex preparation or instrumentation, the system used a custom smartphone-based application and detected all analytes within five minutes, underscoring its viability for use in resource-limited settings. Additionally, MOF-based sensing has substantial potential for use wearable formats. A screen-printed electrochemical platform using a flexible silk-polyurethane composite film was reported for the detection of *E. coli* [140]. Gold-nanoparticle-modified electrodes enabled detection at concentrations as low as 0.12 CFU/mL, and the platform retained its performance after repeated bending. This system has strong applicability in flexible and wearable diagnostic devices.

A wearable, biocompatible fluorescent cotton fabric functionalized with nano-silver/europium aggregates and sodium alginate was developed for dual-mode detection of bacteria (*E. coli* and *S. aureus*) and heavy metals via fluorescence quenching [141]. The material demonstrated good biocompatibility in NIH3T3 cell tests, combined wearability, antibacterial properties, and rapid dual-mode detection, making it a promising platform for on-the-go sensing in environmental and health applications.

### 5.2. Data Processing with AI/ML for Signal Interpretation

The integration of artificial intelligence (AI) and ML into biosensing platforms significantly enhances signal interpretation, enabling more accurate, rapid, and autonomous analysis of complex sensing data [142]. In smartphone-based and wearable biosensors, AI/ML algorithms process optical, electrochemical, or fluorescence data to filter noise, recognize patterns, and quantify analytes with high precision [143]. Techniques such as principal component analysis, support vector machines, and neural networks are frequently used to classify targets, deconvolute overlapping signals, and manage multiplex data from sensor arrays [144]. This data-driven approach improves detection reliability, especially in real-world matrices where environmental variability and user inconsistencies are common. Advanced optical and electrochemical biosensors produce complex datasets that demand intelligent interpretation. AI and ML enable real-time signal analysis, reduce false positives, and improve specificity by learning from large datasets [145,146]. These tools are transforming raw signals into actionable insights for environmental and food safety applications.

From 2019 to 2024, advances in nanobiosensors and AI-enabled systems have driven improvements in pathogen detection and multidrug resistance profiling [147]. These systems combine diverse modalities electrochemical, photothermal, acoustic with deep learning algorithms to enable real-time classification and therapeutic decision-making. In one study, AI was integrated with photonic biosensors to classify bacterial species based on sensor-generated wavelength data. A decision tree classifier was employed to classify bacterial species based on wavelength data generated from photonic sensor simulations [148]. Preprocessing steps included univariate analysis, kernel density estimation, box plot evaluation, feature selection, and outlier removal. The optimized model achieved a classification accuracy of 70.27%, with performance validated via a confusion matrix. These findings highlight the potential of AI-driven signal interpretation to enhance the precision and efficiency of photonic biosensing for bacterial infections.

An AI-powered biosensing framework was developed for pathogen detection in complex food and water environments. An AI-powered biosensing framework was developed for rapid and automated identification of bacteria in food and agricultural water [149]. A deep learning model was trained on augmented images of bacteriophage-tagged bacterial patterns and fine-tuned on mixed cultures. Despite being trained only on lab-cultured samples, the model achieved 80–100% accuracy when applied to real-world water samples with unseen environmental noise, enabling pathogen prediction in under 5.5 h. This demonstrates the strong generalization capability of AI for interpreting complex biosensing data and its potential for real-time microbial water quality monitoring.

Moreover, a novel AI-assisted biosensing strategy a sensing method using artificial intelligence transcoding was developed for rapid and multiplex detection of viable foodborne pathogens. This method uses programmable polystyrene microspheres to visually encode different bacteria, generating distinct optical signals detectable under standard microscopy [150]. A customized computer vision algorithm, trained via ML, interprets these signals to accurately identify pathogen type and count. The system enables amplification-free detection below 10^2^ CFU/mL and distinguishes live from dead bacteria using phage-guided targeting, offering a fast, sensitive, and specific tool for food safety monitoring.

Another study emphasized AI integration for comprehensive food quality assessment. Integrating artificial intelligence with biosensors offers a promising solution for objective, rapid, and comprehensive food quality evaluation [151]. This approach aggregates diverse food quality data and applies AI-driven models to enhance detection accuracy, minimize human error, and enable real-time monitoring. The study underscores the transformative role of AI-assisted biosensing in food safety while discussing future opportunities and challenges for its practical implementation.

**Figure 11:**
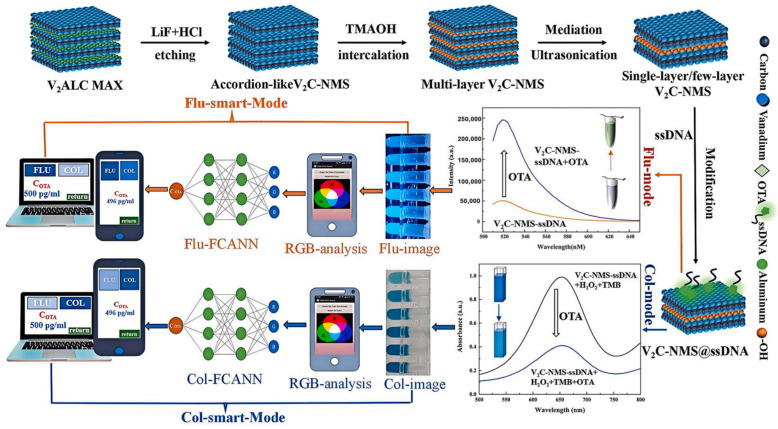
Fabrication strategy of a four-mode nanobiosensor. V_2_C-NMS enables dual-mode OTA detection via fluorescence recovery and colorimetric signal reduction upon ssDNA displacement. Signal changes were quantified and used to train a fully connected artificial neural network (FCANN) for rapid OTA concentration analysis. Copyright 2025 Elsevier [152].

To detect ochratoxin A (OTA) in food, a four-modal MXene-based nano-biosensor system was developed using V_2_C nanosheets and a fully connected artificial neural network (FCANN) trained on RGB signal data. The system uses V_2_C MXene nanosheets with high surface area and peroxidase-like activity for sensitive OTA detection, achieving limits of detection as low as 6.77 pg/mL [152]. A fully connected artificial neural network (FCANN) was trained on RGB data from the biosensor’s outputs, enabling on-site or remote prediction of OTA concentrations within seconds (Figure 11). This AI-assisted approach enhanced detection accuracy and speed, with strong agreement across sensing modes and recovery rates between 95.33% and 105.79%, demonstrating high reliability for real-sample analysis.

Moreover, Wu et al. reported a rapid and accurate detection of multiple pathogens is vital for food safety, disease control, and environmental monitoring, yet traditional methods are limited by complexity and equipment requirements. Optical biosensors offer a promising alternative due to their portability, speed, and multiplexing capabilities. This work reviews recent progress in optical biosensing for multi-pathogen detection, covering core techniques such as colorimetry, fluorescence, SERS, and SPR [153]. It also highlights advancements enabled by microfluidics, nucleic acid amplification, and nanomaterials. While current systems show strong potential, integrating AI and ML for signal interpretation remains a key future direction to enhance accuracy, automation, and real-time decision-making in complex detection environments. AI and ML are also being used for nanosensor design and environmental health monitoring. ML models improve cancer diagnostics and enable rapid, scalable data interpretation for food contaminant analysis, pathogen identification, and nanomaterial toxicity assessment [154].

## 6. Challenges and Opportunities

### 6.1. Stability, Reusability, Cost-Effectiveness

Although MOF-based biosensors have demonstrated exceptional optical and sensing capabilities, their transition from laboratory research to real-world application remains constrained by three interlinked challenges long-term stability, reusability, and cost-effectiveness [155]. Overcoming these limitations is crucial for advancing MOFs from promising experimental materials to commercially viable biosensing platforms.

Stability is the most critical factor determining the operational lifetime of MOF-based sensors. Many MOFs are structurally sensitive to environmental stressors, especially in aqueous or humid conditions, where hydrolysis or ligand displacement can lead to framework collapse [156]. This vulnerability is amplified in real-world matrices containing salts, organic matter, or fluctuating pH levels. The problem becomes more pronounced when sensors incorporate delicate biorecognition elements such as enzymes, antibodies, or nucleic acids, which require narrow temperature and pH ranges to remain functional [157]. For wearable MOF sensors, additional stress factors such as repeated mechanical bending, sweat exposure, and temperature variation during prolonged use can accelerate performance deterioration [158]. Therefore, enhancing intrinsic framework stability through hydrophobic ligand design, post-synthetic modification, or composite encapsulation is essential.

Reusability directly impacts both sensor economics and environmental sustainability. A reusable biosensor reduces per-test cost, minimizes material waste, and enables long-term monitoring without the need for frequent replacement [159]. However, repeated use introduces mechanical fatigue, fouling of active sites, and gradual loss of sensitivity due to surface contamination or irreversible structural changes. Notably, recent work with L-glutamic-acid-based MOFs (L-Glu-M, M = Co, Ni, Cu) has shown progress in this area [160]. These MOFs combined moderate to high surface areas (102.1, 83.7, and 71.0 m^2^/g) with antimicrobial activity against *E. coli* and *S. aureus*, good blood compatibility (≤4% hemolysis, except Cu variant), and high cytocompatibility (~90% viability at 25 μg/mL) [161]. More importantly, they retained the same level of NH_3_ vapor sensing performance across at least five regeneration cycles, indicating that careful material design can deliver both chemical robustness and functional persistence.

Cost-effectiveness remains an equally decisive factor for large-scale adoption. MOF synthesis can involve expensive metal precursors, intricate ligand designs, and energy-intensive processes such as solvothermal synthesis, which hinder scalability [162]. Furthermore, incorporating rare or toxic metals raises both economic and regulatory concerns. Commercial translation particularly in the wearable or disposable biosensor market requires synthesis routes that are low-cost, high-yield, and compatible with mass-production techniques such as inkjet printing, roll-to-roll coating, or polymer embedding [163]. Some recent studies illustrate how rational material choice can balance performance and cost. For example, a zinc-based MOF (Zn-TCPE) exploited its water instability and aggregation-induced emission (AIE) to enable a “turn-off–on” fluorescence response for rapid tobramycin detection in food and water [164]. This design eliminated the need for complex functionalization steps while maintaining high selectivity for aminoglycoside antibiotics. Similarly, a manganese-based MOF achieved ultrasensitive detection of cardiac troponin I, with a limit of detection of 10.0 fg/mL and broad linear range [159], while a La(III)-MOF-Ag nanoparticle FRET system detected microRNA-155 with femtomolar sensitivity, demonstrating diagnostic potential for cancer screening. In another example, hollow-core microstructured optical fiber (HC-MOF) biosensors offered high refractive index sensitivity and a wide detection range for bovine serum albumin, while using a simple, reusable design [165].

Future directions should therefore focus on integrating stability-enhancing modifications with cost-conscious fabrication methods [166]. Strategies may include ligand functionalization to increase hydrophobicity, incorporation of stabilizing polymers or coatings, and selection of earth-abundant metals to reduce raw material costs. Equally important is the development of straightforward regeneration protocols such as solvent rinsing, thermal treatment, or mild chemical washing that can restore sensor performance without damaging the MOF structure. Addressing these challenges in tandem will pave the way for MOF-based biosensors that are chemically and mechanically robust, reliably reusable, and affordable for both high-resource and resource-limited settings [167].

### 6.2. Bridging Lab-Scale and Real-World Application

While numerous MOF-based optical biosensors have demonstrated excellent sensitivity and selectivity in laboratory conditions, translating these systems to real-world applications remains a major hurdle [168]. Complex sample matrices, varying environmental conditions, and the need for minimal sample preparation often reduce sensor performance outside controlled settings. Furthermore, challenges such as sensor integration with portable devices, user-friendly interfaces, and field-deployable formats must be addressed to move from proof of concept to practical implementation. Bridging this gap requires interdisciplinary strategies encompassing engineering, material science, and systems-level design.

Scalability remains a significant limitation in the commercialization of MOF-based wearable sensors, particularly due to the high production costs and uncertainty around consumer acceptance. For successful market integration, products must not only be cost-effective but also user-friendly, safe, and require minimal effort to operate [169]. However, many current sensor designs still demand complex preparation steps, such as pre-use calibration and incubation in conditioning solutions, which are impractical for wearable applications. Additionally, gradual signal deviation over time can compromise analyte accuracy, necessitating frequent recalibration and further hindering widespread adoption [170]. These challenges hinder scalability and adoption, though ongoing research into calibration-free and more robust sensor designs offers promising opportunities to overcome these barriers and enable large-scale deployment.

Translating MOF-based optical biosensors from laboratory research to real-world applications presents both significant opportunities and notable challenges [171]. On one hand, the growing demand for rapid, sensitive, and user-friendly diagnostics particularly in the wake of pandemics like COVID-19 creates a favorable landscape for portable, point-of-care (POC) devices. Optical biosensors leveraging MOFs offer unique advantages, including high surface area, tunable structures, and versatile functionalization, making them well-suited for detecting pathogens with high sensitivity and specificity.

Techniques such as fluorescence sensing, SERS, and plasmonic approaches further enhance their analytical performance. However, moving beyond proof-of-concept systems requires overcoming key challenges: ensuring sensor stability under variable environmental conditions, simplifying sample processing for non-specialist use, reducing fabrication costs, and validating performance with complex real-world samples like bodily fluids or environmental matrices. Moreover, regulatory approval, reproducibility across batches, and user acceptability remain critical hurdles. Nonetheless, with continued advances in materials science, miniaturization, and AI-assisted data interpretation, MOF-based optical biosensors hold strong potential to transition into practical tools for public health surveillance, food safety, and environmental monitoring.

### 6.3. Opportunities in Multimodal Sensing and MOF-Hybrids

The future of MOF-based biosensing lies in the development of advanced platforms that integrate multiple detection modalities and functional hybrid materials. Combining optical sensing with electrochemical, magnetic, or thermal responses can enhance detection accuracy, enable cross-validation, and facilitate multiplexed analysis [172]. Moreover, hybridizing MOFs with nanomaterials such as quantum dots, plasmonic nanoparticles, or two-dimensional (2D) materials can significantly improve signal transduction, stability, and biocompatibility. Such innovations open new avenues for real-time, point-of-care diagnostics, and environmental surveillance with enhanced robustness and adaptability [173].

The future of MOF-based biosensing lies in the development of advanced platforms that integrate multiple detection modalities and functional hybrid materials. Combining optical sensing with electrochemical, magnetic, or thermal responses can enhance detection accuracy, enable cross-validation, and facilitate multiplexed analysis [174]. Moreover, hybridizing MOFs with nanomaterials such as quantum dots, plasmonic nanoparticles, or 2D materials can significantly improve signal transduction, stability, and biocompatibility. Such innovations open new avenues for real-time, point-of-care diagnostics and environmental surveillance with enhanced robustness and adaptability.

Although this subheading focuses on hybrid sensing and multimodality, it is worth noting that one of the key future opportunities is designing MOF-based wearable sensors with improved morphology, biocompatibility, and biodegradability, particularly for in vivo health diagnostics [175]. For these sensors to function effectively on the human body particularly on sensitive areas like the skin they must exhibit not only biocompatibility, but also mechanical flexibility and chemical stability, without interfering with diagnostic results or causing harm.

Additionally, safe removal after use is essential to prevent potential side effects, especially if any component remains in the body or malfunctions during in vivo applications [176]. These requirements pose design and engineering challenges, such as ensuring durability against mechanical stress and chemical changes within biological environments. Despite these hurdles, next-generation MOF-based materials with enhanced biocompatibility, self-degradability, or smart release mechanisms offer a future direction for developing safe, efficient, and disposable wearable biosensors tailored for real-time health monitoring.

## 7. Conclusions and Future Perspective

MOFs have emerged as highly promising materials for the development of advanced optical biosensors, owing to their exceptional tunability, large surface areas, and versatile chemical functionalities. This review has showcased the remarkable progress in employing MOFs across various optical sensing modalities such as fluorescence, chemiluminescence, and colorimetry for sensitive, selective, and rapid detection of pathogens and environmental contaminants. Innovations including MOF hybridization with nanomaterials, integration into microfluidic and wearable devices, and the advent of label-free and smartphone-compatible platforms underscore their potential to transform decentralized diagnostics and environmental monitoring. Despite these advances, several challenges must be addressed to facilitate real-world application. The stability of MOFs under physiological and environmental conditions, reproducibility in large-scale manufacturing, and cost-effectiveness remain significant hurdles. Additionally, regulatory frameworks and standardized validation protocols need to evolve to support the translation of these technologies from the laboratory to commercial markets. Overcoming these obstacles will be critical for broad adoption and impact.

Looking forward, the future of MOF-based optical biosensors lies in multidisciplinary efforts to enhance material robustness, biocompatibility, and functional diversity. Even with all the progress made, a number of thorny issues still linger. For instance, the stability of MOFs when exposed to different temperatures, pH levels, or UV light can be unpredictable, and scaling up production while keeping quality consistent is far from straightforward. Cost considerations also remain tricky, especially when trying to make devices accessible outside well-funded labs. On top of that, clear regulatory guidelines and standardized validation protocols are still lagging behind, which makes it harder to move from promising prototypes to something that can actually be used in clinics or field settings.

1. Looking forward, a few directions seem particularly worth exploring though, admittedly, none of them are simple fixes. These are described as follows. Greener, scalable MOF synthesis: There is a real push toward eco-friendly methods that use less energy and fewer harsh solvents. If successful, this could make industrial-scale production not only more sustainable but also more practical for widespread deployment.
2. AI and digital integration: Pairing MOF sensors with machine learning or AI systems could allow real-time signal analysis or even the ability to detect multiple analytes simultaneously. Still, these setups might be challenging to implement outside high-tech labs, so careful design and validation will be key.
3. Regulatory and point-of-care readiness: Sensors need standardized testing and safety checks to meet the rules for clinical, food safety, or environmental applications. Without this, even the most sensitive device may never see actual use.
4. Operational robustness: Developing MOFs that can reliably handle environmental stressors, humidity, heat, or light exposure would go a long way toward making these sensors field-ready.
5. Miniaturized, multimodal devices: Compact, low-cost sensors that can measure several things at once could be game-changing, especially in remote or resource-limited locations. However, balancing sensitivity with simplicity is often easier said than done.
6. Functional diversity and biocompatibility: Expanding the range of detectable targets, from microbes to volatile compounds, while keeping them compatible with biological samples, remains a delicate engineering challenge.
7. Opportunities abound in developing multimodal sensing platforms that combine multiple optical transduction methods, enabling improved accuracy and reliability. Furthermore, coupling MOF sensors with digital technologies such as artificial intelligence and real-time data analytics offers exciting prospects for smart, autonomous monitoring systems. By addressing current limitations and leveraging emerging trends, MOF-based biosensors are poised to play a vital role in advancing global health, food safety, and environmental sustainability.

## Figures and Tables

**Table 1:** Summary of MOF-based optical biosensors.

Design Strategy	Target Microorganism/Analyte	Detection Method	Advantages	Ref.
MOF with hairpin DNA for gated dye release	Brucella (wild vs. vaccine strain)	Fluorescence	DNA-specific release; low cross-reactivity	[45]
Zn-MOFs with modifiable chemistry	PSA	Fluorescence	Enhanced stability and selectivity	[46]
Cu-MOF with antigen and click-reaction signal amplification	Aflatoxin B1	Fluorescence	Strong proximity-triggered signal amplification	[54]
CD/Co-MOF nanocoral (ratiometric probe)	Alkaline Phosphatase (ALP)	Fluorescence	Dual-signal output; low background noise	[56]
MIL-53(Al)-NH_2_ with FcMBL and aptamer-functionalized beads	*Bacillus cereus*	Fluorescence	Dual recognition; fast detection	[61]
Au NPs@ZIF-MOF functionalized with aptamers	*S. aureus*	Fluorescence	Wide dynamic range; built-in signal calibration	[62]
Au/Ir@Cu/Zn-MOF with anti-*S. aureus* antibodies	*S. aureus*	Immunoassay	High sensitivity; multifunctional	[63]
Ru(bpy)_3_^2+^@Zr-MOF with dsDNA and SGI	ATP (viable bacterial marker)	Fluorescence	Real-time detection; smartphone compatible	[64]
Au@CuMOF and Au@PbMOF functionalized with DNA probes	*S. typhimurium* and *L. monocytogenes*	Electrochemical	Dual-pathogen detection; high selectivity; broad range	[65]

**Table 2:** Summary of MOF-based biosensors for pathogen detection.

Materials	Target Microorganisms	Method	LOD (µg/L)	Linear Range	Ref.
Zr-MOF + DNA aptamer	*E. coli* O157:H7	Fluorescence	–	–	[70]
MOFs + AuNPs + antibodies	SARS-CoV-2	Colorimetric	–	–	[71]
MOFs + lectins	Candida albicans	SERS	–	–	[72]
Fe-MOF@SalmpYZU47	*S. enterica*	Colorimetric	5.5	1.0 × 10^2^ to 1.0 × 10^8^ CFU/mL	[73]
MOF + PTS	*E. coli* O157:H7	Visual	265	–	[74]
GO + Cu–MOF	*M. pneumoniae*, *L. pneumophila*	Electrochemical	0.001	1 pg/mL to 100 ng/mL	[75]
CoFe-MOFs@Nafion	*Salmonella*	Electrochemical	69	1.38 × 10^2^ to 1.38 × 10^8^ CFU/mL	[76]
CoFe-MOFs + MWCNTs + AuNPs	*Salmonella*	Electrochemical	1445	1.04 × 10^4^ to 1.04 × 10^8^ CFU/mL	[77]
MCOF + AuNPs + AIEgens	*S. typhimurium*	Colorimetric/fluorescent	500/5	–	[78]
Cu-MOF	CRP	Colorimetric/fluorescent	0.04/0.240	–	[79]
Zn-MOF	HER2 (non-microbial)	Fluorescence	0.12	–	[80]
UiO-66-NH_2_ MOF + MB + TMB	let-7a, miRNA-21	Electrochemical	0.0022, 0.005	–	[81]
Porphyrinic COFs + AgNPs	CRP	Photoelectrochemical	–	–	[82]
Polyaniline@Ni-MOF	HCV RNA	Electrochemical	0.0048	1 fM to 100 nM	[87]
Zr-MOF + methylene blue	GBM exosomes	Electrochemical	0.0041	9.5 × 10^3^ to 1.9 × 10^7^ particles/μL	[87]
Pt-COF nanozyme + Chromotrope 2R	*L. monocytogenes*, *S. typhimurium*	Colorimetric	0.655, 0.805	–	[89]
CoFe-MOFs + graphene + Au–NH_2_	*Salmonella*	Electrochemical	60	2.4 × 10^2^ to 2.4 × 10^8^ CFU/mL	[90]
Ultrathin MOF-NSs	Multiple bacterial DNAs	Fluorescence	0.009	–	[91]
Fe-MIL-88NH_2_ + PtNPs	*Salmonella*	Colorimetric	46.5	–	[92]
Cu-MOF/PEDOT:PSS	*E. coli* O157:H7	Electrochemical	3.7	3 × 10^2^ to 3 × 10^8^ CFU/mL	[93]
Enzyme-loaded ZIF-8	*E. coli* O157:H7	Colorimetric	0.5	–	[94]
MIL-88@TcP	*S. typhimurium*	Colorimetric	84	–	[95]
ZIF-8 + FLS + CRISPR/Cas12a + RAA	*S. typhimurium*	Fluorescence	65	–	[96]
Cu-MOF + streptavidin	*E. coli*	Colorimetric	1	16 to 1.6 × 10^6^ CFU/mL	[97]
Pt@ZIF-8	*S. typhimurium*	Colorimetric	5.5	10^1^–10^4^ CFU/mL	[98]
MOF@B(OH)_2_ + Ni mesh	*Salmonella*	Fluorescence	9.4	–	[99]
PtNPs + Co/Zn-MOF + MWCNTs	*S. typhimurium*	Electrochemical	47	1.3 × 10^2^ to 1.3 × 10^8^ CFU/mL	[100]
MOF-based micromotors	*E. coli*	Theranostic	–	–	[101]

**Table 3:** Summary of MOF-based optical biosensors for environmental monitoring.

Materials	Target Analytes	Methods	LOD (µg/L)	Real-World Application Challenges	Ref.
Zr-based MOF	Cu^2+^, Pb^2+^, Hg^2+^	Fluorescence	<2	Humic substances, turbidity quenching fluorescence; fouling of sensing sites in natural waters	[108]
P1@BMOF	Cu^2+^	Fluorescence	12.71	UV/temperature induced degradation of azobenzene; limited stability outdoors	[109]
Eu@UiO-MOFs	Cd^2+^	Fluorescence	114	Defect-rich sites prone to hydrolysis at high humidity or pH extremes	[110]
ZIF-67	Cr(VI)	Evanescent	1	Biofilm growth on fiber tip reduces light throughput; requires cleaning	[111]
SM-1	Ag^+^, Cd^2+^, Hg^2+^	Fluorescence	-	Unclear durability under repeated adsorption desorption in field use	[112]
MOF-5	Pb^2+^	Plasmonic	0.5	Ionic strength variations affect plasmonic shifts; turbidity interference	[113]
NH_2_-MIL-101(Fe)-mAb	Pb^2+^	Fluorescence	9.51	Antibody denaturation at high temperatures; cold-chain requirements	[114]
CoPc-PT-COF@Cu-MOF	Cr^3+^	PEC	7.54 × 10^−7^	High background ion interference; PEC components sensitive to fouling	[115]
Bi_2_CuO_4_	Cd^2+^	Electrochemical	2.25 × 10^−6^	Cross-contamination between analytes; electrode surface fouling	[116]
Cu-MOF	Glyphosate	Fluorescence	5.58	Suspended particles scatter light; binding site blockage in field runoff	[117]
ZIF-8@Cellulose	Dichlorvos	Colorimetric	0.29	Enzyme leaching, reduced activity in humid storage	[118]
Tb-BTC MOF	*E. coli*	Fluorescence	0.003	Antibody degradation with temperature/UV; matrix background fluorescence	[119]
ZrPr-MOF	*S. typhimurium*	Colorimetric	0.037	Paper background color interference; aptamer storage stability	[120]
CAU-17/Bi_2_S_3_	3-O-C10-HL	Optoelectronic	1.12 × 10^−4^	Salinity changes affect aptamer binding; marine biofouling	[121]
ZIF-8	H_2_O_2_	NIR-induced	-	Dust, waxes affect coating uniformity; outdoor weather effects	[122]
AuNPs/HKUST-1	VOCs	LSPR	-	VOC mixture overlap; humidity impact on refractive index	[123]
Urease@ZIF-8	Urea	Refractive index	6006	Enzyme instability in heat; long-term wet storage challenges	[124]
UFD-DEC	Urea	Colorimetric	7207.2	Hydrogel dehydration or overhydration alters response	[125]
MOF-CMOS	CO_2_	Colorimetric	26,000	Skin oils/sweat contamination; long-term adhesion to skin	[126]
Cr(III)-MOF-NPs	Morphine	Fluorescence	4.76 × 10^−2^	Autofluorescent backgrounds in biological samples	[127]
Tb-BTC MOF	Hemoglobin	Optical	-	MXene oxidation over time; interference from complex fluids	[128]

## Abbreviations

2D	Two-Dimensional
3-O-C10-HL	N-(3-oxodecanoyl)-L-homoserine lactone
AAS	Atomic Absorption Spectroscopy
ABTS	2,2′-Azino-bis(3-ethylbenzothiazoline-6-sulfonic acid)
AChE	Acetylcholinesterase
AI	Artificial Intelligence
AIA	2-Aminoisonicotinic Acid
AIEgen	Aggregation-Induced Emission Luminogen
ALP	Alkaline Phosphatase
ATP	Adenosine Triphosphate
Au NPs	Gold Nanoparticles
CD	Carbon Nanodot
CFU	Colony-Forming Unit
mL	Milliliter
CMOS	Complementary Metal–Oxide–Semiconductor
COF	Covalent Organic Framework
Cr(VI)	Hexavalent Chromium
MOF	Metal–Organic Framework
Cu-MOF	Copper-Based Metal–Organic Framework
DNA	Deoxyribonucleic Acid
DPV	Differential Pulse Voltammetry
dsDNA	Double-Stranded Deoxyribonucleic Acid
*E. coli*	*Escherichia coli*
ELISA	Enzyme-Linked Immunosorbent Assay
FcMBL	Fragment Crystallizable Mannose-Binding Lectin
FRET	Fluorescence Resonance Energy Transfer
GBM	Glioblastoma Multiforme
GO	Graphene Oxide
HCR	Hybridization Chain Reaction
HER2	Human Epidermal Growth Factor Receptor 2
hCG	Human Chorionic Gonadotropin
HPLC	High-Performance Liquid Chromatography
HRP	Horseradish Peroxidase
IFE	Inner Filter Effect
IoT	Internet of Things
LOD	Limit of Detection
LSPR	Localized Surface Plasmon Resonance
miRNA	MicroRNA
ML	Machine Learning
MXene	Transition Metal Carbide
MWCNT	Multi-Walled Carbon Nanotube
NIR	Near-Infrared
NMOF	Nanoscale MOF
NS	Nanosheet
OTA	Ochratoxin A
PCR	Polymerase Chain Reaction
PEDOT:PSS	Poly(3,4-ethylenedioxythiophene):poly(styrenesulfonate)
PET	Photoinduced Electron Transfer
POCT	Point-of-Care Testing
PSA	Prostate-Specific Antigen
PtNP	Platinum Nanoparticle
PTS	Pregnancy Test Strip
RAA	Recombinase-Aided Amplification
RNA	Ribonucleic Acid
*S. aureus*	*Staphylococcus aureus*
SARS-CoV-2	Severe Acute Respiratory Syndrome Coronavirus 2
SERS	Surface-Enhanced Raman Scattering
SGI	SYBR Green I
SPR	Surface Plasmon Resonance
TcP	Tetraphenylporphyrin
TMB	3,3′,5,5′-Tetramethylbenzidine
UFD-DEC	Urease–Fe-cdDNA Dual-Enzyme Cascade
UV–vis	Ultraviolet–Visible Spectroscopy
VOC	Volatile Organic Compound
ZIF	Zeolitic Imidazolate Framework
